# *In Vivo* Ligands of MDA5 and RIG-I in Measles Virus-Infected Cells

**DOI:** 10.1371/journal.ppat.1004081

**Published:** 2014-04-17

**Authors:** Simon Runge, Konstantin M. J. Sparrer, Charlotte Lässig, Katharina Hembach, Alina Baum, Adolfo García-Sastre, Johannes Söding, Karl-Klaus Conzelmann, Karl-Peter Hopfner

**Affiliations:** 1 Gene Center and Department of Biochemistry, Ludwig-Maximilians University Munich, Munich, Germany; 2 Max von Pettenkofer-Institute, Gene Center, Ludwig-Maximilians University Munich, Munich, Germany; 3 Center for the Study of Hepatitis C, Laboratory of Virology and Infectious Disease, The Rockefeller University, New York, New York, United States of America; 4 Department of Microbiology, Department of Medicine, Division of Infectious Diseases and Global Health and Emerging Pathogens Institute, Icahn School of Medicine at Mount Sinai, New York, New York, United States of America; 5 Center for Integrated Protein Science Munich, Munich, Germany; McMaster University, Canada

## Abstract

RIG-I-like receptors (RLRs: RIG-I, MDA5 and LGP2) play a major role in the innate immune response against viral infections and detect patterns on viral RNA molecules that are typically absent from host RNA. Upon RNA binding, RLRs trigger a complex downstream signaling cascade resulting in the expression of type I interferons and proinflammatory cytokines. In the past decade extensive efforts were made to elucidate the nature of putative RLR ligands. *In vitro* and transfection studies identified 5′-triphosphate containing blunt-ended double-strand RNAs as potent RIG-I inducers and these findings were confirmed by next-generation sequencing of RIG-I associated RNAs from virus-infected cells. The nature of RNA ligands of MDA5 is less clear. Several studies suggest that double-stranded RNAs are the preferred agonists for the protein. However, the exact nature of physiological MDA5 ligands from virus-infected cells needs to be elucidated. In this work, we combine a crosslinking technique with next-generation sequencing in order to shed light on MDA5-associated RNAs from human cells infected with measles virus. Our findings suggest that RIG-I and MDA5 associate with AU-rich RNA species originating from the mRNA of the measles virus L gene. Corresponding sequences are poorer activators of ATP-hydrolysis by MDA5 *in vitro*, suggesting that they result in more stable MDA5 filaments. These data provide a possible model of how AU-rich sequences could activate type I interferon signaling.

## Author Summary

RIG-I-like receptors (RLRs) are helicase-like molecules that detect cytosolic RNAs that are absent in the non-infected host. Upon binding to specific RNA patterns, RLRs elicit a signaling cascade that leads to host defense via the production of antiviral molecules. To understand how RLRs sense RNA, it is important to characterize the nature and origin of RLR-associated RNA from virus-infected cells. While it is well established that RIG-I binds 5′-triphosphate containing double-stranded RNA, the *in vivo* occurring ligand for MDA5 is poorly characterized. A major challenge in examining MDA5 agonists is the apparently transient interaction between the protein and its ligand. To improve the stability of interaction, we have used an approach to crosslink MDA5 to RNA in measles virus-infected cells. The virus-infected cells were treated with the photoactivatable nucleoside analog 4-thiouridine, which is incorporated in newly synthesized RNA. Upon 365 nm UV light exposure of living cells, a covalent linkage between the labeled RNA and the receptor protein is induced, resulting in a higher RNA recovery from RLR immunoprecipitates. Based on next generation sequencing, bioinformatics and *in vitro* approaches, we observed a correlation between the AU-composition of viral RNA and its ability to induce an MDA5-dependent immune response.

## Introduction

The retinoic acid inducible gene I (RIG-I)-like receptor (RLR) proteins are key players in innate immunity and act by recognizing viral RNA (vRNA) in the cytosol. The RLR family consists of the members retinoic acid inducible gene I (RIG-I), melanoma differentiation associated protein 5 (MDA5), and laboratory of genetics and physiology 2 (LGP2) [1]–[3]. *In vitro* studies have shown that RIG-I and MDA5 recognize the majority of viruses in a complementary manner. While many negative-strand RNA viruses like rabies and influenza viruses are predominantly sensed by RIG-I, picornaviruses are predominantly recognized by MDA5. The observed preferences are, however, unlikely to be exclusive and the exact role of LGP2 still needs to be investigated [4]–[9]. In case of MDA5, a minor contribution to recognition of measles, rabies, vesicular stomatitis and Sendai virus has been reported [10]–[13].

The RLR proteins belong to the DExD/H-box ATPases sharing a central ATP-dependent helicase domain and a C-terminal regulatory domain (RD) that is responsible for initial RNA binding. In addition, RIG-I and MDA5 possess N-terminal tandem caspase activation and recruitment domains (CARDs) that are responsible for downstream signaling transduction [2], [14], [15]. Several crystal structures of RIG-I have shown that, in the absence of virus, the protein exists in an auto-inhibited state where the RD domain folds back to the CARDs, thereby shielding them from the cytosol. Upon viral infection and initial vRNA binding, the protein undergoes large conformational changes leading to the interaction with the mitochondrial associated signaling protein (MAVS) [16]–[19]. This leads to the activation of a downstream signaling cascade and finally to the induction of type I interferon (IFN) expression and the establishment of an anti-viral state. Although the exact nature of RLR ligands is not yet fully understood, several studies report that RIG-I preferentially binds to relatively short (between 25 to 1000–2000 bp) 5′-triphosphate double-stranded RNAs (5′-triphosphate dsRNA) like those of Sendai virus (SeV) defective interfering (DI) particles [20]–[23]. In contrast, MDA5 seems to have a preference for long (more than 1000–2000 bp) dsRNA stretches [24], [25]. Upon binding to dsRNA, MDA5 is thought to cooperatively form polar helical filaments leading to association with MAVS and activation of the downstream signaling cascade [26]–[28].

Viruses have developed numerous strategies to evade the immune system. For instance, viruses of the paramyxovirus family (e.g. measles, parainfluenza, Sendai and Nipah viruses) encode V inhibitor proteins that specifically bind to MDA5 and LGP2, but not always to RIG-I [29]–[31]. By determining the structure of MDA5 in complex with parainfluenza virus V-protein, we previously showed that the viral protein unfolds the ATPase domain of MDA5. This leads to the disruption of the MDA5 ATP-hydrolysis site and prevents RNA bound MDA5 filament formation [32].

One of the remaining key questions in this field is how RLR proteins are able to distinguish between self and non-self RNA in the cytosol. Recently, several studies showed that 5′-triphosphate RNA is not the only RNA ligand for RIG-I. Specific poly U/C-rich regions within certain viral genomes seem to contribute to efficient recognition by the protein [33], [34]. In case of MDA5, it is not known which features of vRNA are required in order to induce an immune response. Expression of subgenomic and subgenic RNA from parainfluenza virus 5 (PIV5) indicated that MDA5 recognizes a specific region within the L mRNA [35]. For picornaviruses, it is speculated that MDA5 binds to long dsRNA that represents replicative intermediates composed of the positive genome and the negative antigenome [36]. These studies were, however, based on *in vitro* transfection experiments and it has so far not been possible to isolate a natural RNA ligand for MDA5 directly from virus-infected cells.

In this study we combined different methods, including RNA-protein crosslinking and deep sequencing, to investigate *in vivo* RNA ligands for RLR proteins from virus-infected cells. Based on the crosslinking we were able to co-purify immunostimulatory RNA in a RIG-I and MDA5 dependent manner from measles virus (MeV)-infected cells. Deep sequencing and bioinformatics analysis revealed that RIG-I and MDA5 bind RNA of positive polarity originating from the L gene of the MeV genome. In addition, RIG-I binds to the 5′ ends of genomic and antigenomic RNAs, which probably represent 5′-triphosphate RNA, and are therefore not recognized by MDA5. Furthermore, we showed that RIG-I, but not MDA5, binds RNA of negative polarity, indicating that MDA5 does not efficiently recognize the MeV genome. Based on bioinformatics analysis, we observed a correlation between MDA5-enriched RNA sequences and the AU content and this was confirmed by *in vitro* transcription assays. In summary, we report the isolation of MDA5-associated RNA from virus-infected cells and the discovery of *in vivo* occurring activating viral RNA ligands for MDA5.

## Results

### 4-thiouridine treatment and 365 nm UV light exposure lead to improved RLR-associated RNA recovery from virus-infected cells

**Figure 1:**
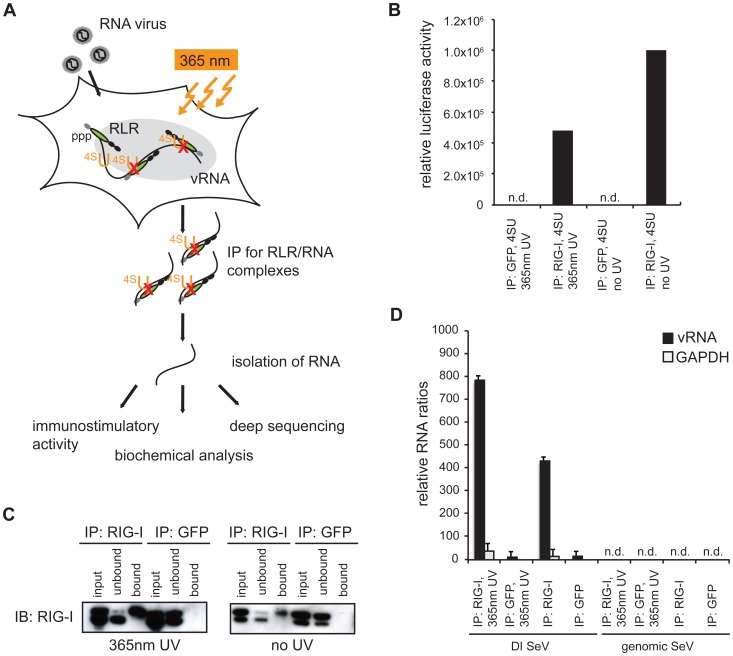
Validation of crosslinking and immunoprecipitation of RLR/RNA complexes from 24 h virus-infected cells. **A:** Schematic representation of the experimental procedure for characterization of RLR-associated RNA molecules. **B:** Immunostimulatory activity of RNA from RIG-I and control (GFP) crosslinking samples in comparison to non-crosslinking immunoprecipitates. **C:** Western blot analysis of crosslinked and non-crosslinked RIG-I and control (GFP) pull-down experiments. **D:** Comparison of RNA recovery levels by quantitative PCR analysis of RIG-I-associated RNA from SeV-infected cells (n = 3). n.d. = not detectable.

Several *in vitro* studies showed that MDA5 preferably recognizes long dsRNA stretches [24], [25]. However, it is still unclear if the protein has a preference for specific RNA sequences. The main reason for this may lie in the weak interaction between the protein and its ligand resulting in very poor RNA levels that co-purify from MDA5 immunoprecipitates. In order to address this problem, we established an RNA-protein crosslinking approach adapted from the PAR-CLIP (**P**hoto**a**ctivatable-**R**ibonucleoside-Enhanced **C**ross**l**inking and **I**mmuno**p**recipitation) methodology [37]. With this approach, we intended to improve RNA recovery from RLR immunoprecipitates in the context of a viral infection. For validation of the method, we compared the crosslinking approach with a conventional pull-down technique previously used for the identification of SeV DI particles as potent RIG-I inducers [20]. We infected A549 human lung carcinoma cells with SeV at a high multiplicity of infection (MOI) in the presence of 4-thiouridine (4SU) and allowed infection to occur over 24 h. A part of the cells was then exposed to 365 nm UV light and endogenous RIG-I was immunopurified (**Figure 1a**). The recovered RNA was isolated and subjected to quantitative PCR (qPCR) analysis and immunoactivity experiments. The data indicate that treatment of cells with 4SU and exposure to 365 nm UV light lead to a reduction of immunostimulatory activity of RIG-I-associated RNA to 50% (**Figure 1b**). However, the results of qPCR analysis showed that the crosslinking approach yields a quantitatively improved RNA recovery, with an increase of 50% in SeV DI particles in comparison to the non-crosslinking approach (**Figure 1c and d**). Furthermore, we confirmed that treatment of cells with the photoreactive nucleoside does not affect cell viability or virus replication (data not shown). Taken together, our data indicate that the crosslinking technique is a promising tool to study *in vivo* occurring RNA ligands for RLR proteins.

**Figure 2:**
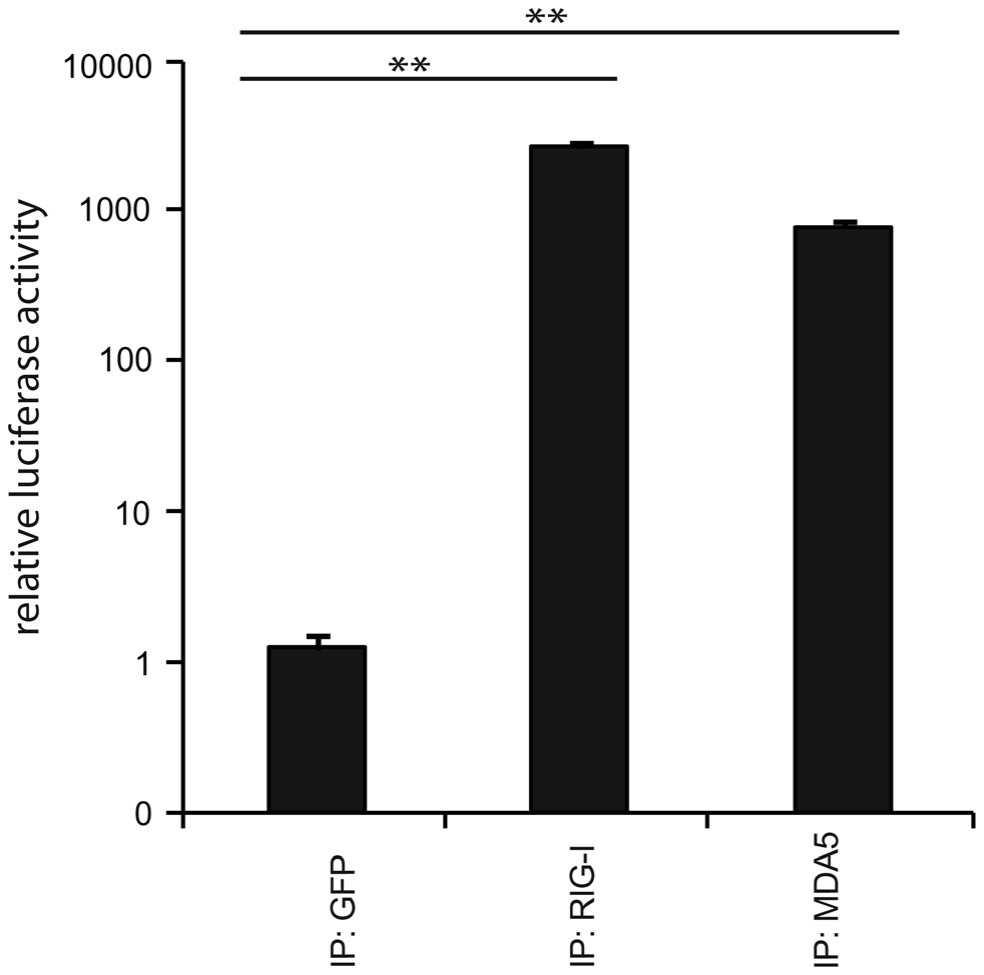
Immunoprecipitation of RLR-associated RNA from 24 h MeV infections. Validation of immunostimulatory activity of RNA from RIG-I, MDA5, and GFP immunoprecipitates upon transfection into 293T ISRE-FF reporter cells (n = 3, ** P<0.01).

Next, we validated the crosslinking approach on cells that were infected with a variety of viruses, including negative-stranded (−) RNA viruses (MeV [38] and rabies [39]) and positive-stranded (+) RNA viruses (Encephalomyocarditis virus (EMCV [40]) and Mengo virus [41]). In all cases, we infected A549 cells at an MOI of 1.0 in the presence of 4SU. Cells were crosslinked 24 h post infection (hpi) and RIG-I and MDA5 were immunopurified. The recovered RNA was subjected to immunoactivity experiments. Based on the data, we concluded that immunoactive RNA was co-purified in a RIG-I- and MDA5-dependent manner from MeV-infected cells. This induction was significant in comparison to the negative control (**Figure 2**). In the case of RIG-I-associated RNA, we obtained an immunostimulatory effect that was 2600-fold higher in comparison to the control. For MDA5, we observed an 800-fold induction. The data show that the approach yields RIG-I- and MDA5-specific immunoactive RNA from MeV-infected cells in a RIG-I- and MDA5-dependent manner. Although we detected significant immunostimulatory activity for RLR-associated RNAs from MeV-infected cells, the experimental set up is currently unsuitable for the isolation of RLR RNA ligands from the other viruses (**Figure S1**). The reason for this may lie in the heterogeneity and the need for precise timing of viral replication cycles or in the efficiency of 4SU incorporation and crosslinking. Utilization of this technique for other viruses may require adjustment of parameters, such as the time points of 4SU addition, crosslinking and harvesting after infection.

### Deep sequencing reveals regions within the measles virus genome recognized by RIG-I and MDA5

Based on the above-mentioned results, we focused our studies on MeV, which belongs to the order of *Paramyxoviridae*. MeV has a single-stranded RNA genome of negative polarity consisting of 15,894 nucleotides. It comprises six non-overlapping genes, which are flanked by small terminal non-coding regions known as leader (*le*) and trailer (*tr*) sequences. These sequences serve as promoter regions during viral replication and transcription [42], [43]. While the replication of the genome and antigenome is performed in a continuous process, viral transcription is carried out in a sequential manner, giving rise to an mRNA gradient declining in the 3′ to 5′ direction (**Figure S2**), as previously published [44]. Since (−) RNA virus polymerases eventually fail in transcription termination, they generate, in addition to monocistronic mRNAs, numerous alternative RNA species including read-through transcripts, such as leader-N, bi- or tricistronic mRNAs [45]. Furthermore, replication can give rise to abortive replication products and DI RNA with large internal deletions or copy-back genomes [46]. Due to the complex RNA composition of a virus-infected cell, the analysis of specific RNA ligands for RLR proteins is challenging.

In order to shed light on the exact nature of RIG-I and MDA5-associated RNAs derived from MeV-infected cells, we performed a deep sequencing analysis on isolated RNA species from co-immunopurifications with antibodies against endogenous RIG-I and MDA5. As a control, we used an antibody against GFP (GFP protein was not present). The MeV strain used for the studies presented here was a recombinant measles virus rescued from cDNA with the exact sequence of the Schwarz vaccine strain (Genbank AF266291.1) [38].

**Figure 3:**
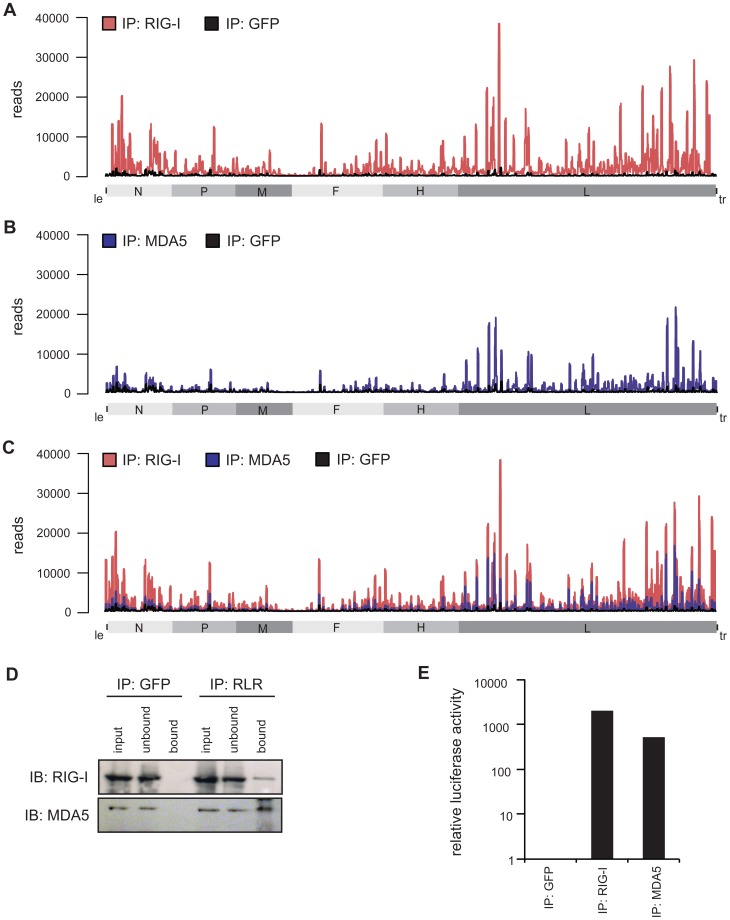
Deep sequencing analysis of RLR-associated RNA from MeV-infected cells. **A–C:** RNA from RIG-I pull-down (red), MDA5 pull-down (blue), and control (GFP) pull-down (black) from MeV-infected cells were subjected to Illumina deep sequencing analysis. Obtained sequencing reads are mapped to their position on the viral antigenome. The x-axis corresponds to all possible 15,894 positions in the MeV antigenome and the y-axis shows the number of reads at the respective position. (*A*) RIG-I-associated sequences in comparison to the control mapped to the viral antigenome. (*B*) MDA5-associated sequences in comparison to the control mapped to the viral antigenome. (*C*) Overlay of RIG-I associated and MDA5-associated sequences. **D:** Western blot analysis of RIG-I and MDA5 immunopurification in comparison to the GFP pull-down. **E:** Validation of immunostimulatory activity of RNA from RIG-I, MDA5, and GFP immunoprecipitates upon transfection into 293T ISRE-FF reporter cells.

Obtained sequences were mapped to the MeV antigenome and the relative abundances of these sequences between RIG-I pull-down, MDA5 pull-down, and GFP pull-down were compared. Analysis of the reads showed that RIG-I and MDA5 bind to similar regions within the L gene-derived RNAs. In addition, RIG-I, but not MDA5, binds to RNAs derived from the 3′ and the 5′ ends of the MeV genome (**Figure 3a and b**). These regions probably represent *le* or *tr*RNA generated in the course of replication or transcription. Additionally, internal genomic and antigenomic sequences found in the pull-downs could potentially originate from MeV DI particles [46]–[51]. To address this question, we performed a PCR analysis of RLR libraries in which we specifically amplified copyback DI RNA of MeV [47]–[49]. Indeed, we detected copyback DI particles not only in the RIG-I pull-down but also within RNA recovered from MDA5 immunoprecipitates (**Figure S3**). We did not find DIs in the GFP control pull-downs.

Consistent with previous work, the higher copy numbers of reads indicate that RIG-I binds MeV RNA with higher affinity than MDA5 [11]. This observation is in good agreement with the increased immunostimulatory activity of isolated RNA from RIG-I pull-down samples in comparison to MDA5. Regarding the immunostimulatory activity, RIG-I-associated RNA gives a 4-fold higher induction in comparison to MDA5-associated RNA (**Figure 3d and e**).

### Analysis of deep sequencing data reveals remarkable differences in the strand-specificity of RIG-I and MDA5

**Figure 4:**
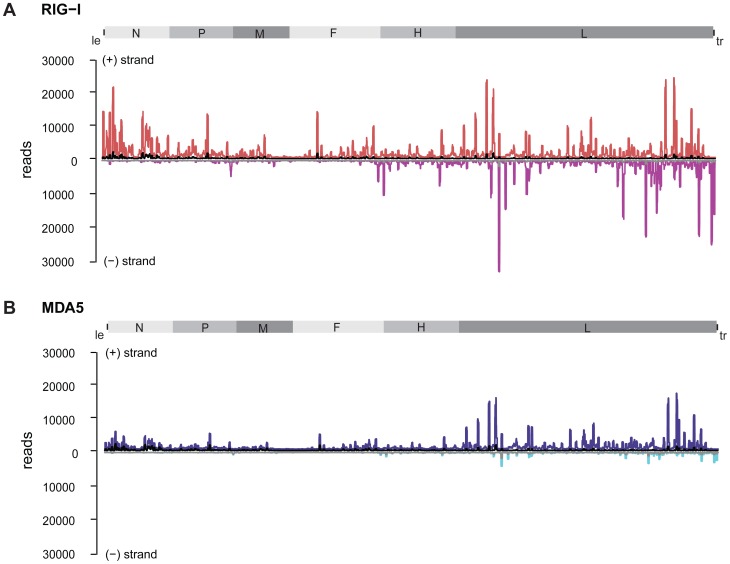
Strand separation of sequencing libraries into (+) and (−) MeV RNA. RNA deep sequencing libraries were generated based on the strand-specific mRNA sample preparation protocol from Epicentre. The Epicentre protocol encompasses sequential ligation of 5′ and 3′ adapters to RNA molecules, thus preserving strandness information. **A:** RIG-I-associated sequences in comparison to the control mapped to the viral antigenome. (+) RNA is shown in red; (−) RNA is shown in magenta; the control library is shown in black and grey. **B:** MDA5-associated sequences in comparison to the control mapped to the viral antigenome. (+) RNA is shown in blue, (−) RNA is shown in cyan; the control library is shown in black and grey.

Based on the protocol used for cDNA library preparation, sequencing reads could be separated according to their strand orientation. During cDNA synthesis, adaptors were specifically ligated to the 3′ or 5′ ends, thereby keeping the information of strand specificity during the deep sequencing run. Separation of sequences revealed remarkable differences between both protein immunoprecipitations. RIG-I associated RNA sequences of positive polarity, which represent either antigenomic RNA or mRNA transcripts, are enriched in regions close to the 5′ end of the viral antigenome (leader) but also in distinct regions within the L gene. In contrast, sequences of negative polarity, representing the viral genome, are exclusively enriched in the 5′ end of the genome (trailer region) and in regions of the L gene (**Figure 4a**).

Analysis of MDA5-associated RNA revealed that sequences of positive polarity were enriched within the L gene originating from similar regions as (+) RNA from the RIG-I library (**Figure 4b**). In contrast to RIG-I, however, MDA5 did not bind to RNA sequences comprising the 5′ end of the antigenome or leader RNA. Comparison of (−) RNA from RIG-I and MDA5 libraries further revealed that, in contrast to RIG-I, MDA5 did not enrich sequences of negative polarity, including trailer sequences.

According to the analysis of strand specific enrichment, it appears that MDA5 does not bind vRNA of negative polarity that represents the MeV genome. Furthermore, the data evidently rule out the possibility that MDA5 recognizes RNA duplexes of (+) and (−) RNA that might represent replication intermediates, as previously suggested for a positive-strand RNA virus [36]. In fact, the result suggests that MDA5 binds (+) RNA that could either represent mRNA or the MeV antigenome.

To further validate the specificity of the accumulation of RIG-I and MDA5-associated RNA, we calculated specific read enrichments [52] of the RLR libraries compared to the control library (**Figure S4**). Enrichment (greater than 2× compared to the control library) of RIG-I-associated RNA of positive polarity can be found across the whole genome, whereas only few reads of negative polarity are enriched within the N and L segment. In contrast, enriched sequences of MDA5-associated RNA are exclusively present within the L segment of positive polarity, whereas no specific enrichment was observed for (−) RNA.

Based on the data, we observed a good correlation between the deep sequencing analysis and enrichment calculations, indicating that distinct regions within the MeV genome are indeed specifically enriched in a RIG-I- and MDA5-dependent manner in comparison to the control.

### Confirmation of deep sequencing data with quantitative PCR

**Figure 5:**
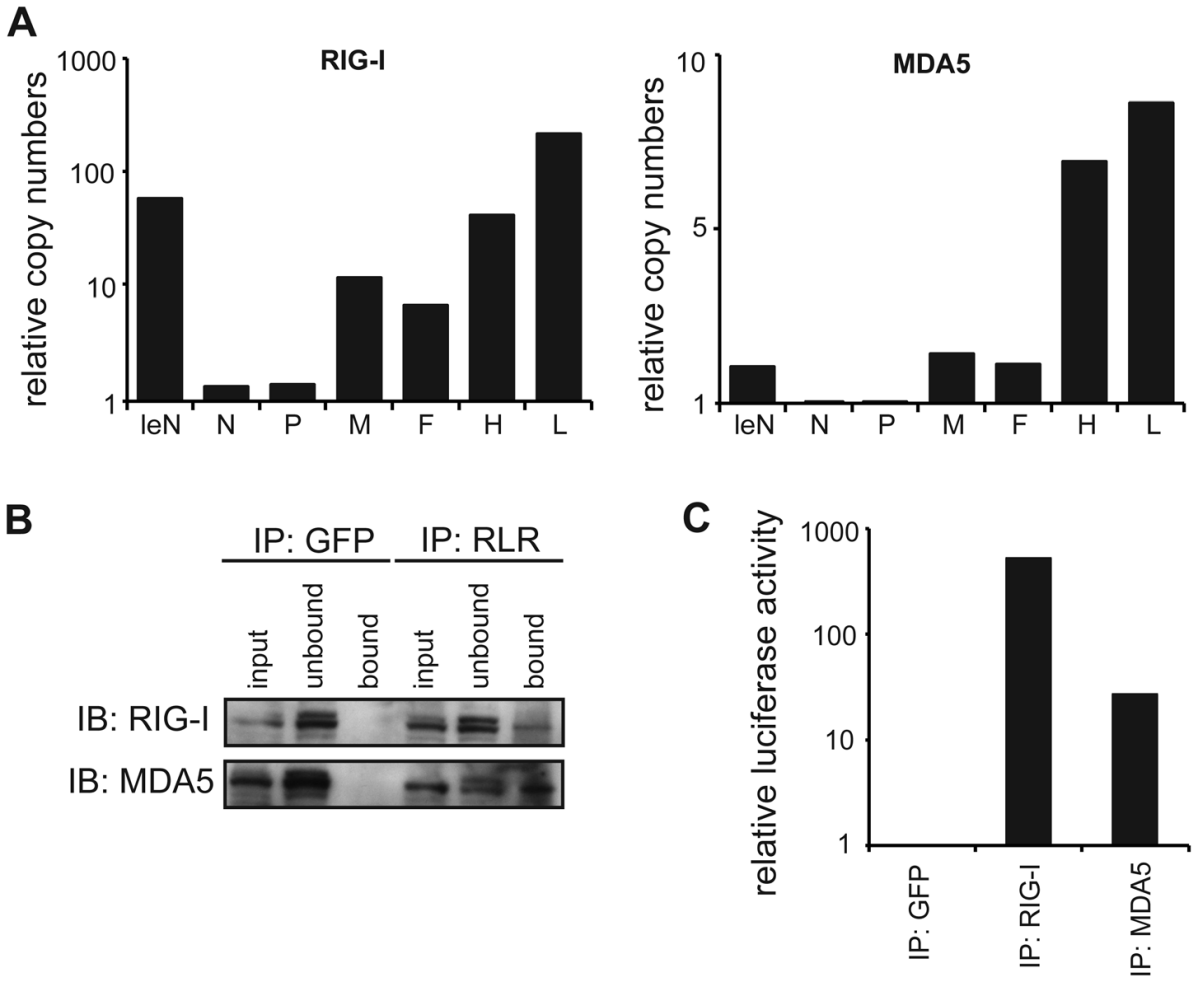
Quantitative PCR analysis of RLR-associated RNA from MeV-infected cells. **A:** Comparison of RNA levels between RIG-I and MDA5 immunoprecipitates for each genomic segment at 24 hpi. Relative RNA ratios were normalized against the control (GFP) library (mean values, n = 2). **B:** Western blot analysis of RLR pull-down experiments in comparison to the GFP pull-down. **C:** Immunostimulatory activity of RLR-associated RNA at 24 hpi.

To independently validate the relative amount of RLR-associated RNA, qPCR amplification was performed. The obtained copy read numbers were normalized to the GFP negative control in order to compare the genomic segments in the RIG-I and MDA5 samples (**Figure 5a**). Analysis of relative abundances confirmed that RIG-I specifically enriches sequences from the 3′ and 5′ regions of the MeV genome, representing either antigenome or viral mRNA. Interestingly, the analysis showed that RIG-I-associated RNA from the genomic 3′ end most likely represents leader read-through transcripts or abortive replication products and not N mRNA. In MDA5 pull-downs, RNA was enriched in the case of the L mRNAs and partly in the case of H mRNAs, while no relevant copy numbers were obtained at other genomic positions. This is in good agreement with the results of the deep sequencing analysis, indicating that MDA5 indeed recognizes RNA originating from the L gene of the MeV genome. Furthermore, comparison of the relative copy numbers between RIG-I and MDA5 revealed remarkable differences between both proteins. The relative abundances in the RIG-I sample were up to 40-fold higher in comparison to MDA5. This observation again indicates that RIG-I has a higher affinity for MeV RNA sequences in comparison to MDA5. Our conclusion is further supported by immunoactivity experiments, where the relative immunostimulatory activity of RIG-I-associated RNA was 20-fold higher in comparison to MDA5 (**Figure 5b and c**).

### Bioinformatics analysis reveals a preferred binding of RIG-I and MDA5 to RNA species with a higher AU composition

**Figure 6:**
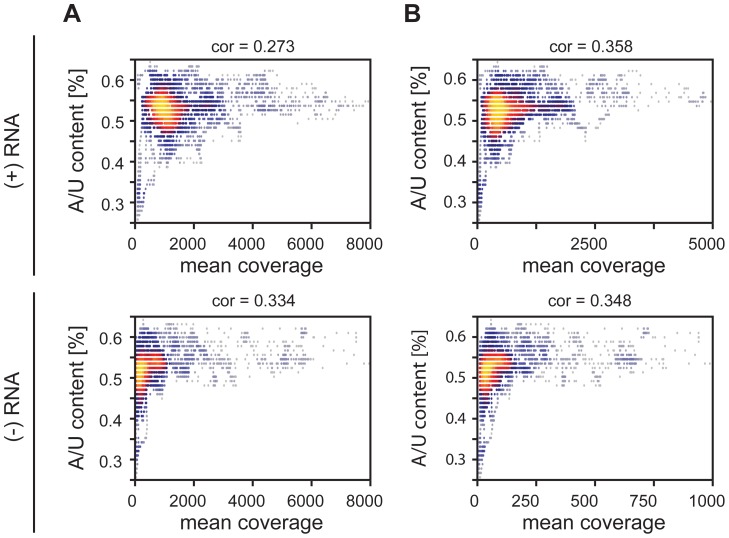
Heatscatter plots of AU content of 201 nucleotide MeV RNA fragments and the fragment's mean coverage. The linear correlation is expressed via the Pearson coefficient. Every dot corresponds to one fragment with its respective AU content and mean coverage within an RLR library. The more yellow the plot, the more data points overlap. **A:** Correlation between AU composition and coverage of RIG-I-associated RNA of positive and negative RNA respectively. **B:** Correlation between AU composition and coverage of MDA5-associated RNA of positive and negative RNA respectively.

To elucidate the exact nature of sequences enriched by RIG-I and MDA5 immunoprecipitations, we conducted a bioinformatics analysis. For this, the complete genome was divided into fragments of size 201 nt with a shifting window of 5 nt. Each sequence was folded *in silico* (RNAfold [53]) and several RNA primary and secondary structure features were analyzed. The analyzed parameters were set in relation to the mean coverage of sequencing reads from RIG-I and MDA5 pull-down experiments. Heat scatter plots indicate that sequences rich in AU correlate with a high mean coverage of sequencing reads in both the RIG-I (cor = 0.273, cor = 0.334) and MDA5 (cor = 0.358, cor = 0.348) libraries (**Figure 6a and b**). These data suggest that RIG-I and MDA5 preferably bind to AU-rich sequences originating from the viral genome. Although we further analyzed a variety of secondary structure parameters, including paired nucleotides and bulges, we did not see any other relevant correlation with the mean coverage of sequencing reads (**Figure S5 and Figure S6**).

### Confirmation of deep sequencing analysis by immunoactive experiments on *in vitro* transcripts

**Figure 7:**
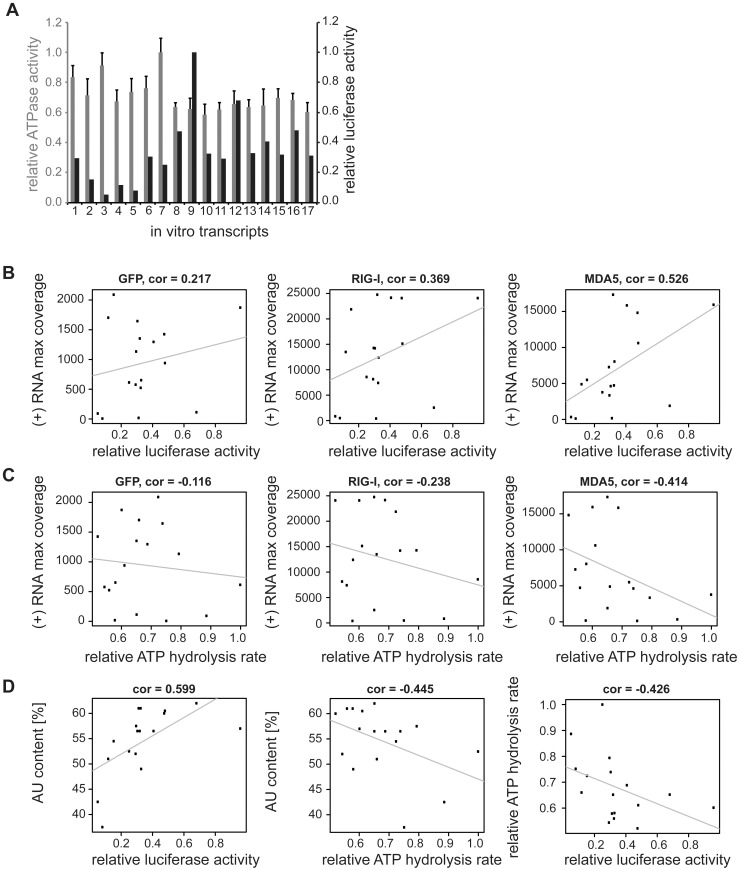
Analysis of *in vitro* transcribed RNA of the measles virus genome. Sequences were generated according to deep sequencing data. The transcripts were either transfected into 293T ISRE-FF reporter cells in order to validate the immunostimulatory potential or ATPase hydrolysis experiments were performed in presence of recombinant *m*MDA5. **A:** Comparison of relative luciferase activities (black) and relative ATPase activities (grey) of *in vitro* transcribed RNAs (n = 3 and n = 2 respectively, values were normalized to the highest mean value of each replicate). **B:** Pearson correlation between (+) RNA maximal coverage and relative luciferase activity. **C:** Pearson correlation between (+) RNA maximal coverage and relative ATPase activity. **D:** Correlation analysis between AU content and luciferase or ATPase activity, and between ATPase and luciferase activity.

To further confirm the obtained sequencing data, we generated 17 single-stranded, 200 nucleotide long *in vitro* transcripts (IVTs) covering different regions of the MeV antigenome (**Table S1**). RNAs were double-dephosphorylated in order to ensure that 5′-triphosphate groups were removed. For immunoactivity experiments IVTs were transfected into 293T ISRE-FF reporter cells. The stimulatory effect revealed a correlation of high read numbers from deep sequencing analysis and high stimulatory activity of the IVT sequences (**Figure 7**). According to the immunostimulatory experiment, we observed increased immunostimulatory activities for transcripts 8, 9, and 12 (**Figure 7a**). These transcripts correspond to regions at the 5′ end of the L gene, which is also the region with the highest copy numbers of reads (**Figure 3**). In general, IVTs representing regions within the L gene have higher immunostimulatory activity in comparison to the upstream genomic segments. This is in good agreement to the deep sequencing analysis. Furthermore, calculated Pearson correlations showed that the best correlation between maximal numbers of sequencing reads and the immunostimulatory activity of RNA transcripts can be found in the MDA5 sequencing data (cor = 0.526), while RIG-I and GFP samples showed less correlation (cor = 0.369 and cor = 0.217) (**Figure 7b**). In order to find a possible explanation for the different immunostimulatory potentials of IVTs, several characteristics of the transcripts were analyzed *in silico*. The obtained data revealed that the immunostimulatory potential correlates with the AU content of IVTs (cor = 0.599) (**Figure 7d**), which is consistent with the results from the deep sequencing analysis. Visualization of transcripts on an Agilent bioanalyzer RNA chip indicates that no higher-order structures due to the sequence composition were formed that might explain differences in immunostimulatory activity (data not shown).

In order to get a more general conclusion about the contribution of the AU content to the immunostimulatory potential of RNAs, *in vitro* transcripts from Mengo virus (**Table S3**) were tested for their immunostimulatory activity. The transcripts were generated according to the protocol for MeV RNA sequences. We again observed a correlation (cor = 0.583) of the AU content of the tested sequences and their immunostimulatory potential (**Figure S 7a and b**). These data are consistent with the *in vitro* analysis of MeV RNA sequences indicating that the AU composition of RNA might play a general role in activating RLR signaling.

### *In vitro* transcripts with a low proportion of AU strongly stimulate ATP hydrolysis by MDA5

Finally, we asked whether the ATP hydrolysis activity of MDA5 correlates with the immunostimulatory potential of the tested IVTs. We measured the ATP hydrolysis rate of recombinant mouse MDA5 in the presence of RNA transcripts (**Figure 7** **and Figure S8**) and observed a negative correlation between the maximum number of sequencing reads in the MDA5 library and the ATP hydrolysis rate (cor = −0.414, **Figure 7c**). Analysis of the *in vitro* data revealed that AU-rich sequences lead to a decrease in ATP hydrolysis activity of MDA5 (cor = −0.445). Furthermore, the ATP hydrolysis rate negatively correlates with the immunostimulatory potential of RNA transcripts (cor = −0.426) (**Figure 7d**). This result suggests that the ATPase hydrolysis activity of MDA5 is not correlated to the binding and the immunostimulatory potential of the RNA transcripts and could therefore provide a model of RNA recognition by the protein. The data are consistent with previous work on MDA5 filament formation upon dsRNA binding [26], [27]. In structural and biophysical studies, Berke *et al* showed that ATP hydrolysis by MDA5 causes filaments to disassemble, perhaps by inducing translocation along the RNA or triggering a conformational change in the protein. According to our data, this may explain the observed inverse correlation between the immunostimulatory activity of IVTs and their potential to induce the ATPase activity of MDA5.

## Discussion

Until now, *in vivo* RLR ligands were poorly understood and a naturally occurring MDA5 ligand could only be purified indirectly by immunoprecipitation of LGP2:RNA complexes from virus-infected cells overexpressing LGP2 [54]. By applying a combination of RNA-protein crosslinking, immunoprecipitation of endogenous proteins and RNA deep sequencing analysis, we were able to investigate RLR-associated RNA from MeV infected cells. We compared our results to the empty GFP antibody control resembling a previously published immunoprecipitation strategy [20].

Our approach reveals that MDA5 preferentially binds measles virus RNA of positive polarity, whereas RIG-I additionally binds to (−) sense RNA within the trailer region as well as in the adjacent L gene. We propose that enriched RNA of positive polarity most likely represents mRNA species, since antigenomic RNA is only generated during replication and is immediately packed into nucleocapsids [55]–[57]. For *Mononegavirales*, these RNA-protein complexes are considered inaccessible for cytoplasmic proteins [55], [58] and might not be ligands for RLR proteins unless they become released. We show that, unlike MDA5, RIG-I binds (+) sense RNA originating from not only the L genomic segment, but also from the 3′ end of the MeV genome, which could be either *le*-N read-through transcripts or abortive replication products comprising 5′-triphosphate ends [45], [46]. Furthermore, we hypothesize that RIG-I specific enriched RNA of negative polarity represents abortive replication products also having 5′-triphosphate ends [20]–[23]. Additionally, 5′-copyback DI sequences combining vRNA of positive and negative polarity were found both in RIG-I and MDA5 immunoprecipitates and may contribute to recognition [49].

Bioinformatics analysis and *in vitro* transcription experiments revealed a correlation between AU content and read coverage of the obtained sequences or IVTs, respectively. As shown before [59], this indicates that RNA rich in AU could serve as a putative ligand for RIG-I and MDA5, or in a secondary manner lead to a specific structure that is recognized by both proteins. The slightly weaker correlation of RIG-I associated sequences with their AU content compared to MDA5 bound RNAs could be explained by additional sequences or triphosphate RNAs recognized by RIG-I that originate from regions less rich in AU.

Interestingly, ATP hydrolysis assays performed with recombinant MDA5 and RNA transcripts indicate that the AU content of RNA negatively correlates with the ATP hydrolysis rate of the protein. This inverse correlation between the immunostimulatory potential of RNAs and their capability to stimulate ATP hydrolysis by MDA5 lets us speculate that the ATPase activity might not be necessary for, or even interfere with, the immunoactivity of RNA ligands. Although this observation disagrees with recent findings about the role of ATP hydrolysis in RIG-I oligomerization on 5′-triphosphate dsRNA [60], we assume that MDA5 and RIG-I differ markedly in their mechanical activation and the role of ATP hydrolysis. Our data is supported by results suggesting that MDA5 filament formation is abrogated in an ATP-sensitive manner. By electron microscopy (EM) analysis it was shown that MDA5 filaments disassemble in the presence of ATP, indicating that ATP hydrolysis triggers the translocation of the protein along the dsRNA molecule or reduces the binding affinity, thereby interfering with downstream signaling [26], [27]. In light of the available data in the literature we therefore hypothesize that the ATPase activity of the MDA5 helicase domain contributes to substrate specificity by detaching the protein from low affinity substrates. To further test this hypothesis we generated RIG-I^E373Q^ and MDA5^E444Q^, which are mutated in the “Walker B” ATP hydrolysis motif [61], slowing down or abrogating the ATP hydrolysis activity of the proteins, while preserving formation of ATP complexes. Overexpression of these mutant proteins from transfected plasmids showed a dramatic increase in their immunostimulatory potential in the absence of any viral ligands in comparison to expressed wild-type MDA5 (**Figure S9**). Furthermore, pull-down studies with the RIG-I Walker B mutant revealed an increase in the amount of recovered RNA while their immunostimulatory potential decreased (data not shown). The increased immunostimulation of ATPase deficient RLRs is consistent with the model that RNAs that lead to a reduced ATP-hydrolysis rate are more proficient in immunostimulation, possibly by stabilizing RLR∶RNA complexes. The negative correlation between AU-rich sequences and the ATP hydrolysis rate suggests that MDA5 binds AU-rich RNA in preference to GC-rich RNA. This would lead to a stronger interaction between RNA and MDA5 and result in a higher immunostimulatory signal. In order to test this hypothesis, we performed binding assays with MDA5 and IVTs but we were not able to demonstrate differences in the binding affinities between the different transcripts that might support this theory (data not shown). Finally, we speculate that RNA ligands for RLR proteins could be divided into two classes. The first class would comprise RNA molecules originating from the 5′-triphosphate ends of the genome or antigenome. These molecules could be generated in the course of read-through transcription and abortive replication [45], [46] and could therefore represent preferred ligands of RIG-I, as shown previously [20]. The second class of RNA molecules could be recognized by both receptor proteins. Our data suggest that recognition of these RNAs might occur through the AU composition of sequences [34]. This second class might also prominently include defective interfering (DI) particles generated during MeV replication. For MDA5, however, our deep sequencing data show that the (−) strand portion of the DIs is either relatively short or the fraction of DIs binding to MDA5 is magnitudes lower than the binding to L derived (+) sense RNAs and therefore not easily detectable during sequencing. A more detailed analysis of the deep sequencing data is currently ongoing in order to shed more light on the complex nature of the DIs involved.

It will be interesting to see what types of RNA associate with RIG-I and MDA5 during infections with different viruses and to what extent the AU composition and DI generation contributes to RNA recognition in these types of viruses. In particular, the finding that both RIG-I and MDA5 localize to AU rich regions suggests partially overlapping roles in detection of different viruses. The specificity of RIG-I and MDA5 for certain viruses may lie not only in the detection of 5′-triphosphate by RIG-I, but also in the heterogeneity of viral evasion strategies [62]. Our findings support a model for the recognition of AU-rich sequences by RIG-I and MDA5 from MeV-infected cells. Consistently, we find a similar correlation for *in vitro* transcribed RNA from the Mengo virus genome.

In general, the data support previous experiments indicating that MeV is mainly recognized by RIG-I, while MDA5 seems to play a minor role [4], [5], [13], [63]. It could be possible that RIG-I initially recognizes *le*-N read-through transcripts or abortive replication products containing 5′-triphosphate ends, leading to the activation of the signaling cascade. In a second round of recognition, RIG-I and MDA5 then recognize viral transcripts that are rich in AU. To further test this hypothesis, time dependent experiments need to be carried out.

One feature of the applied crosslinking technique is the introduction of specific T to C transitions at the interaction sites of 4SU-labeled RNA and the protein upon UV light exposure [37]. By identifying these point mutations in the deep sequencing data, one can exactly pinpoint the RNA sequences that interact with the protein of interest. However, our bioinformatics analysis did not reveal significant enrichment of T to C mutations, which could be explained by the rather low incorporation efficiency of the photoreactive nucleoside into viral RNA, consistent with the low incorporation level of 4SU into host RNA. Nevertheless, by increasing the incorporation efficiency in future studies, the identification of point mutants could further narrow down the precise binding sites of RLRs.

In summary, our approach provides a first insight into the molecular basis of vRNA derived from MeV interaction with MDA5 in living cells and reveals a preference for binding of AU-rich regions originated from (+)-sense RNA of the L gene. *In vitro*, these RNA molecules appear to be a poorer stimulator of the ATPase activity of MDA5, and result in more stable MDA5 filaments and support better downstream signaling.

## Materials and Methods

### Cell lines, viruses and antibodies

Infection experiments were carried out in A459 human lung carcinoma cells. HEK 293T ISRE-FF reporter cells (stable expression of firefly luciferase under the control of an interferon stimulated response element) were used for interferon stimulation luciferase reporter gene assays. All cells were maintained in Dulbecco's Modified Eagle Medium supplemented with 2 mM L-glutamine, 1% Penicillin-Streptomycin and 10% FBS (all purchased from Invitrogen). Viruses used for infections were recombinant measles virus with a sequence identical to the vaccine strain Schwarz (AF266291.1.), Sendai virus, Sendai virus defective interfering particles H4 (kindly provided by Dominique Garcin, Geneva, Switzerland), Mengo virus strain pMC0 (kindly provided by Anne Krug, TU Munich, Germany) and EMCV. Primary antibodies to human MDA5 (AT113) and RIG-I (Alme-1) were purchased from Enzo Life Science (Loerrach, Germany). Antibody to GFP (ab1218) was obtained from Abcam (Cambridge, UK). Secondary antibodies were supplied by GE Healthcare (Buckinghamshire, UK).

### Crosslinking and immunoprecipitation of RLR-associated RNA from virus-infected cells

A549 cells were infected with virus with an MOI of 1.0 in the presence of 400 µM 4SU. Infection was allowed to proceed for 24 h and living cells were washed with PBS (10 mM phosphate, 137 mM NaCl, 2.7 mM KCl, pH 7.5) and exposed to 1 J/cm^2^ 365 nm UV light using a photocrosslinker (Vilbert Lourmat). Cells were harvested and incubated in Nonidet P-40 lysis buffer (50 mM HEPES, 150 mM KCl, 1 mM NaF, 10 µM ZnCl_2_, 0.5% NP-40, 0.5 mM DTT, protease inhibitor, pH 7.5) for 10 min on ice. The lysate was cleared by centrifugation and endogenous proteins were immunoprecipitated for 4 h with the respective antibodies (1 µg/mL) bound to protein G Dynabeads (Life Technologies). The beads were washed five times with high-salt wash buffer (50 mM HEPES, 500 mM KCl, 0.05% NP-40, 0.5 mM DTT, protease inhibitor, pH 7.5) and incubated with proteinase K (Thermo Scientific) for 30 min at 55°C. The RNA was isolated by phenol/chloroform/isoamylalcohol extraction and subjected to further analysis.

### Total RNA isolation from virus infected cells

A549 cells were infected with MeV with an MOI of 1. Cells were harvested 24 hpi. Total RNA was isolated according to manufacturer's protocol of the RNeasy Protect Mini Kit (Qiagen) and subjected to Illumina deep sequencing.

### Luciferase transfection assay

Immunoactivity experiments were carried out in 24-well plates. 2.5×10^5^ HEK 293T ISRE-FF reporter cells were transfected with 250 ng of recovered RNA, 500 ng *in vitro* transcripts or 500 ng plasmid DNA using Lipofectamine 2000 (Invitrogen) according to manufacturer's protocol. After 24 h incubation, cells were subjected to immunoactivity experiments using the Dual-Glo luciferase assay system (Promega) according to manufacturer's instructions. The luciferase activity was determined in a 96-well plate reader. Significance of differences in luciferase activity between samples were determined via an unpaired t-test.

### cDNA library preparation and deep sequencing analysis

Isolated RNA was prepared for Illumina sequencing using the mRNA-Seq library preparation kit (Epicentre) according to manufacturer's protocol. To remove ribosomal RNA species from the sequencing libraries a Ribo-Zero rRNA removal kit (Epicentre) was used. Quality of RNA-Seq libraries was validated on a DNA1500 chip for the Bioanalyzer 2100 (Agilent). Sequencing was performed on the Illumina Genome Analyzer in the Gene Center sequencing facility (LAFUGA). Obtained sequences were processed with the FASTX toolkit (http://hannonlab.cshl.edu/fastx_toolkit/) in order to remove adapter sequences and reads with PHRED scores below 30. Remaining sequences were mapped to human and viral genomes by utilization of the Bowtie algorithm [64], allowing maximal one mismatch per unique read. The Bowtie sequence alignments were converted with SAMtools [65] to pileup format, which was subsequently used for further data analysis. Relative sequence abundances were analyzed between RLR pull-down samples and the GFP control. Specific read enrichments were calculated by determining the relative sequence abundance at each position on the genomic segment and calculating the average of the RLR/GFP ratios over a dynamic window of 200 reads. Relative sequence abundances with log_2_ ratios above +1 were defined as significantly enriched in the RLR library.

### Analysis of RNA sequences

RNA secondary structure prediction from measles virus genome or *in vitro* transcripts was performed by utilization of RNAfold from the ViennaRNA package [53] using standard parameter settings. For this purpose, the genome was divided into 201 nt fragments with a shifting window size of 5 nt. The sequences were folded *in silico* and the linear relationship between different data sets was quantified with the Pearson correlation coefficient.

### SYBR green quantitative PCR analysis

DNase treatment of the immunoprecipitated RNAs and qPCR was performed as previously described [66]. The primer pairs used for quantification were identical to those published [67]. For cDNA synthesis a random hexanucleotide mix was used (Roche). Full length MeV vac2 cDNA with a known concentration was used for standard generation. Copy number values obtained for MDA5 and RIG-I were normalized to the control GFP.

### PCR for 5′-copyback defective interfering genome detection

Specific primers for reverse transcription (Roche transcriptor transcriptase) and the subsequent PCR (Biozym Phusion Polymerase) were adapted from Calain et al [47]. PCR products were analyzed on agarose gels and stained with ethidium bromide.

### T7 RNA transcription

Templates were generated for *in vitro* transcription in a PCR adding the T7 promoter sequence (TAATACGACTCACTATA GGG) to the 5′ end of the desired MeV or Mengo virus genomic fragment, respectively (for oligonucleotides see **Tables S2 und S4** respectively). PCR products were subsequently purified on agarose gels. RNA was transcribed using the Ambion Megashortscript T7 Kit according to the manufacturer's protocols. The reaction was incubated overnight at 37°C and RNA was precipitated using LiCl at −20°C for 30 minutes. Afterwards, RNA was subjected to triphosphate digestion using FastAP (Fermentas) according to the manufacturer's instructions and purified on denaturing 8 M urea/10% polyacrylamide gels at 25 mA constant current. Gel slices containing RNA were incubated overnight with 450 µL probe elution buffer (0.5 M ammonium acetate, 1 mM EDTA, 0.2% SDS). Eluted RNA was isolated by phenol/chloroform/isoamylalcohol extraction and precipitated with ethanol.

### ATPase hydrolysis assays

ATPase hydrolysis activity was determined using [γ-P^32^] ATP. Mouse MDA5 was purified as described previously [32] and 1.6 µM of protein was preincubated with 80 nM *in vitro* transcribed RNA for 10 min at room temperature. The reaction was initiated by addition of ATPase hydrolysis buffer (20 mM HEPES, pH 7.5, 150 mM NaCl, 1.5 mM MgCl_2_, and 2 mM DTT) containing 2 mM ATP and 0.2 µCi [γ-P^32^] ATP. The hydrolysis rate was monitored over 1 h and analyzed by thin layer chromatography (TLC).

### Generation of RLR mutants

Sequences encoding full-length human RIG-I with N-terminal FLAG-tag and full-length human MDA5 with N-terminal FLAG-tag were cloned into pcDNA5 FRT/TO (Invitrogen). Mutants (FLAG-RIG-I E373Q and FLAG-MDA5 E444Q) were generated by site directed mutagenesis with PfuUltra (Agilent).

## Supporting Information

Figure S1 **Validation of immunostimulatory activity of RNA from RIG-I, MDA5, and GFP immunoprecipitates upon transfection into 293T ISRE-FF reporter cells** (n = 3, **P<0.01).
(TIF)

Figure S2 **Deep sequencing analysis of total RNA from MeV-infected cells 24 hpi.** RNA was isolated according to manufacturer's protocol of the RNeasy Protect Mini Kit (Qiagen) and total RNA was subjected to Illumina deep sequencing. The data show an mRNA gradient declining in the 5′ to 3′ direction, while RNA of negative polarity has no relevant copy numbers.
(TIF)

Figure S3 **Qualitative PCR analysis of MeV copyback DI RNA.** Following a specific reverse transcription of RNA with a primer binding at the 3′-terminus of the antigenome, 5′-copyback DI genomes were specifically amplified using another primer in the same direction 600 nt downstream. The PCR was afterwards analyzed on agarose gels to separate the amplicons of specific copyback DIs with different length and branching points. The RNA used for these experiments is indicated on the lanes (RIG-I, MDA5 and GFP immunoprecipitates).
(TIF)

Figure S4 **Enrichments in RLR sequencing libraries.** Binary logarithms of RLR to GFP ratios of sequence reads (log_2_([read number RLR/read number GFP] * [total read number GFP/total read number RLR])) were calculated in order to determine specific accumulations within the RLR libraries. Data points with log_2_ ratios above 1 represent sequencing reads that were enriched in comparison to the control (GFP) library. **A:** Enrichments within the whole RIG-I (+) or (−) stranded sequencing library. **B:** Enrichments within the whole MDA5 (+) or (−) stranded sequencing library. **C:** Similar to A and B, but zoomed in view of the enrichments for positive polarity (le)N and L segments. Mean values for RIG-I and MDA5 log_2_ ratios are shown in red and blue, respectively. Standard deviations are represented in grey. The mean coverage of (+) RNA sequences is shown for the RIG-I (red), MDA5 (blue), and GFP (black) libraries below each graph.
(TIF)

Figure S5 **Secondary structure analysis of several features from** ***in silico*** **folded 201 nucleotide MeV RNA fragments and correlation to the fragment's mean coverage within the RIG-I sequencing library.** *In silico* folding was done with RNAfold using standard parameters. The analysis is visualized in heatscatter plots and the linear correlation is expressed via the Pearson coefficient. Every dot corresponds to one fragment with its depicted feature and mean coverage. The more yellow the plot, the more data points overlap. Analyzed RNA features are: number of paired nucleotides, longest paired stretch, number of stem-loops, mean size of stem-loops, number of bulges, mean size of bulges and mean number of paired nucleotides per branch. **A:** Correlation analysis of RNA secondary structure features with the RIG-I associated RNA of positive polarity. **B:** Correlation analysis of RNA secondary structure features with the RIG-I associated RNA of negative polarity.
(TIF)

Figure S6 **RNA secondary structure analysis of several features from** ***in silico*** **folded 201 nucleotide MeV RNA fragments and correlation to the fragment's mean coverage within the RIG-I sequencing library.** Foldings were performed with RNAfold using standard parameters. The analysis is visualized in heatscatter plots and the linear correlation is expressed via the Pearson coefficient. Every dot corresponds to one fragment with its depicted feature and mean coverage. The more yellow the plot, the more data points overlap. Analyzed RNA features are: number of paired nucleotides, longest paired stretch, number of stem-loops, mean size of stem-loops, number of bulges, mean size of bulges and mean number of paired nucleotides per branch. **A:** Correlation analysis of RNA secondary structure features with the RIG-I associated RNA of positive polarity. **B:** Correlation analysis of RNA secondary structure features with the RIG-I associated RNA of negative polarity.
(TIF)

Figure S7 **Analysis of** ***in vitro*** **transcribed RNA of the Mengo virus genome.** Six 201 nt fragments were chosen to include low and high AU content. Transcripts were transfected into 293T ISRE-FF reporter cells in order to validate the immunostimulatory potential. **A:** Relative luciferase activity of transfected RNA (n = 3). **B:** Pearson correlation between (+) RNA maximal coverage and the relative luciferase activity.
(TIF)

Figure S8 **MDA5 ATPase activity assay.** Free phosphate was separated by thin layer chromatography (TLC) and visualized on a Storm PhosphorImager from Molecular Dynamics. The ATPase hydrolysis rate was determined by quantifying free phosphate in comparison to non-hydrolyzed ATP 15 min after adding [γ-P^32^] ATP to the reaction mixture.
(TIF)

Figure S9 **Immunostimulatory activity of overexpressed Walker B mutants RIG-I^E373Q^ and MDA5^E444Q^ compared to wildtype proteins in 293T ISRE-FF reporter cells** (n = 3, ** P<0.01, *** P<0.001).
(TIF)

Table S1 **Sequences of** ***in vitro*** **transcribed MeV RNAs.** The gene annotation with the exact nucleotide position on the MeV genome is shown in brackets.
(DOCX)

Table S2 **Oligonucleotides used for generation of** ***in vitro*** **transcribed MeV sequences.**
(DOCX)

Table S3 **Sequences of** ***in vitro*** **transcribed Mengo RNAs. T**he gene annotation with the exact nucleotide position on the Mengo virus genome is shown in brackets.
(DOCX)

Table S4 **Oligonucleotides used for generation of** ***in vitro*** **transcribed Mengo sequences.**
(DOCX)
