# Supplementary figures and images for: In Vivo Ligands of MDA5 and RIG-I in Measles Virus-Infected Cells
Source: PLoS Pathog. 2014 Apr 17;10(4):e1004081. doi: 10.1371/journal.ppat.1004081 (PMC3990713; doi:10.1371/journal.ppat.1004081)

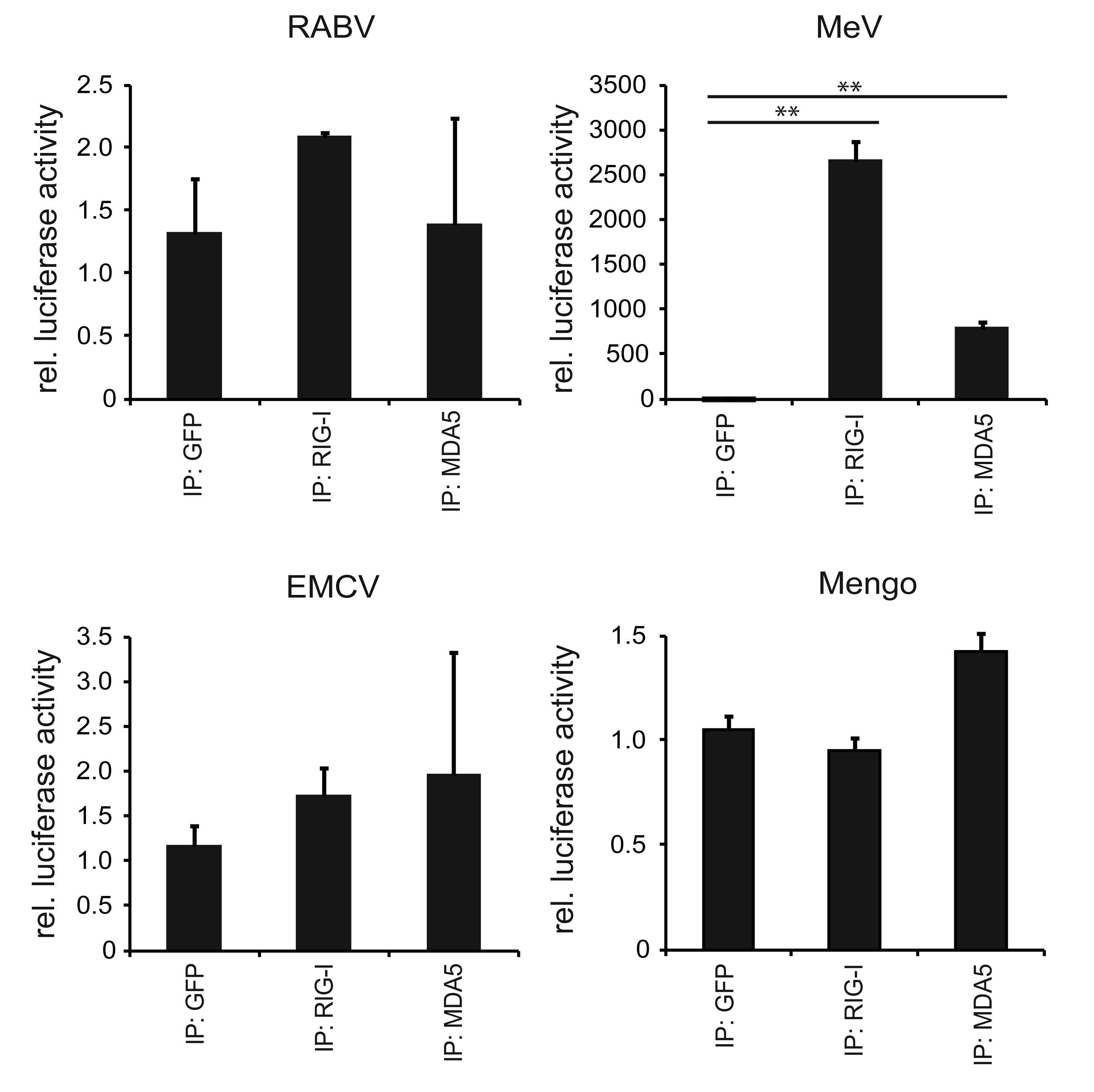

Supplement: Figure S1 — Validation of immunostimulatory activity of RNA from RIG-I, MDA5, and GFP immunoprecipitates upon transfection into 293T ISRE-FF reporter cells (n = 3, **P<0.01). (TIF) [file ppat.1004081.s001.tif]

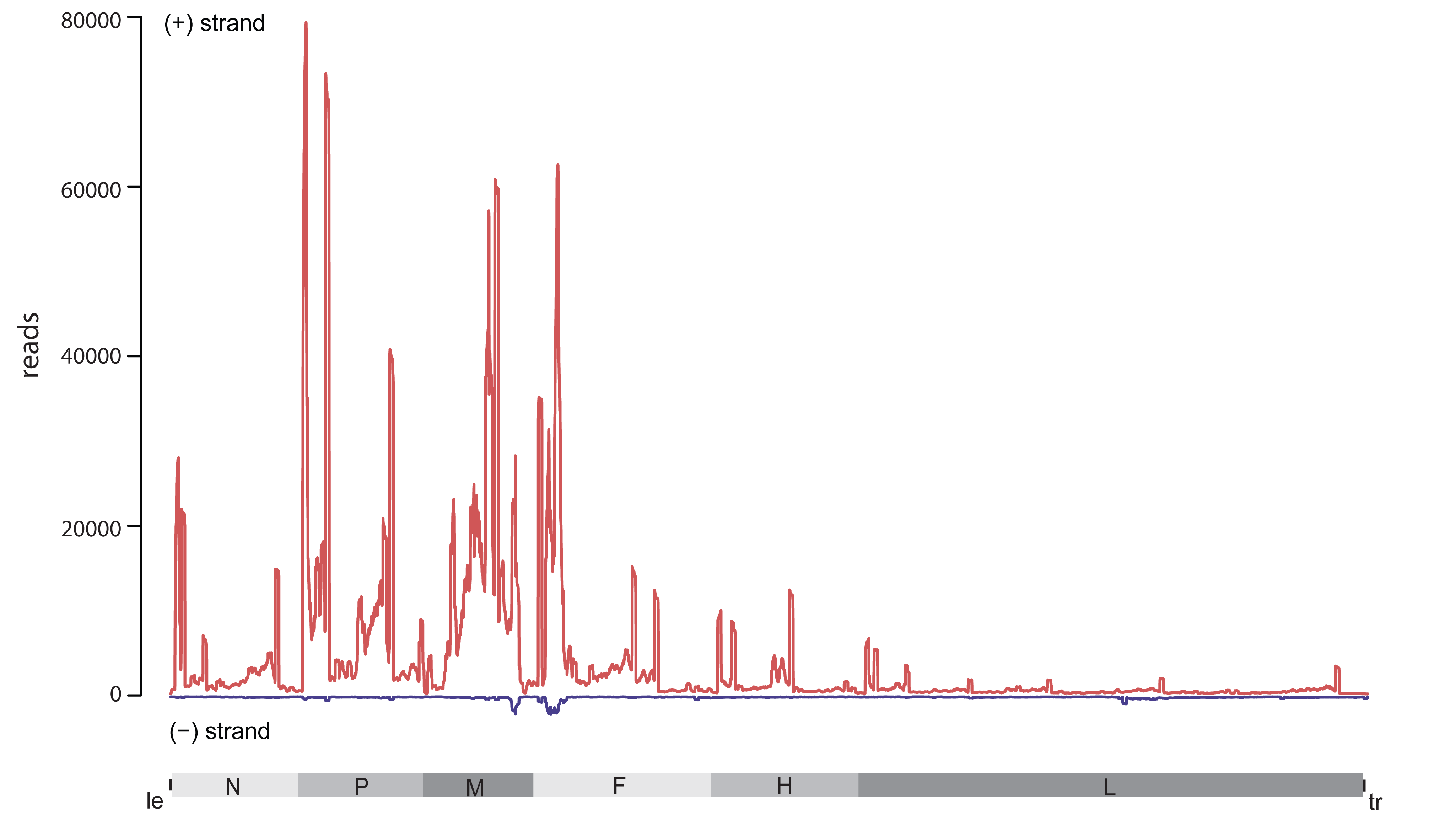

Supplement: Figure S2 — Deep sequencing analysis of total RNA from MeV-infected cells 24 hpi. RNA was isolated according to manufacturer's protocol of the RNeasy Protect Mini Kit (Qiagen) and total RNA was subjected to Illumina deep sequencing. The data show an mRNA gradient declining in the 5′ to 3′ direction, while RNA of negative polarity has no relevant copy numbers. (TIF) [file ppat.1004081.s002.tif]

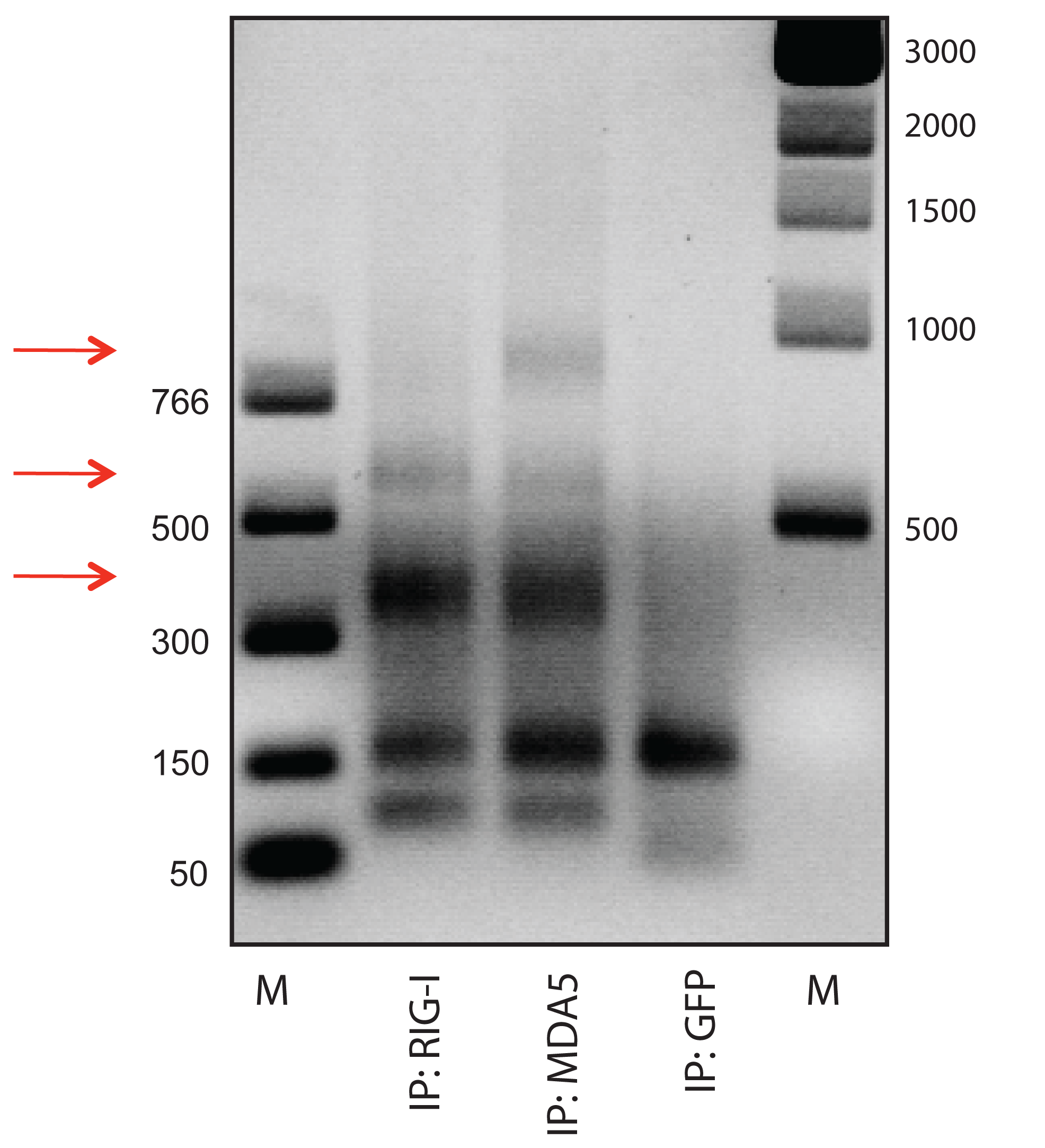

Supplement: Figure S3 — Qualitative PCR analysis of MeV copyback DI RNA. Following a specific reverse transcription of RNA with a primer binding at the 3′-terminus of the antigenome, 5′-copyback DI genomes were specifically amplified using another primer in the same direction 600 nt downstream. The PCR was afterwards analyzed on agarose gels to separate the amplicons of specific copyback DIs with different length and branching points. The RNA used for these experiments is indicated on the lanes (RIG-I, MDA5 and GFP immunoprecipitates). (TIF) [file ppat.1004081.s003.tif]

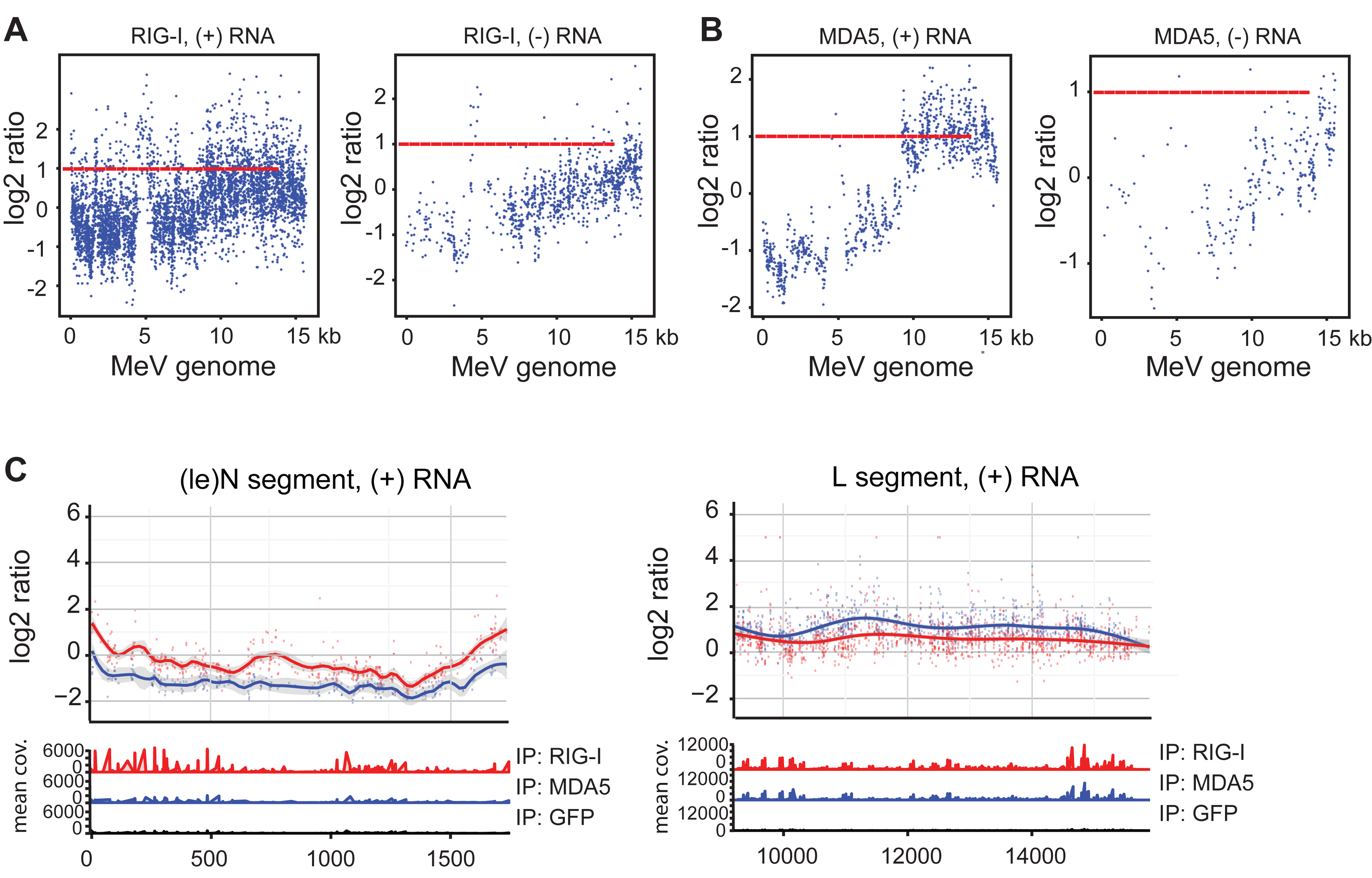

Supplement: Figure S4 — Enrichments in RLR sequencing libraries. Binary logarithms of RLR to GFP ratios of sequence reads (log2([read number RLR/read number GFP] * [total read number GFP/total read number RLR])) were calculated in order to determine specific accumulations within the RLR libraries. Data points with log2 ratios above 1 represent sequencing reads that were enriched in comparison to the control (GFP) library. A: Enrichments within the whole RIG-I (+) or (−) stranded sequencing library. B: Enrichments within the whole MDA5 (+) or (−) stranded sequencing library. C: Similar to A and B, but zoomed in view of the enrichments for positive polarity (le)N and L segments. Mean values for RIG-I and MDA5 log2 ratios are shown in red and blue, respectively. Standard deviations are represented in grey. The mean coverage of (+) RNA sequences is shown for the RIG-I (red), MDA5 (blue), and GFP (black) libraries below each graph. (TIF) [file ppat.1004081.s004.tif]

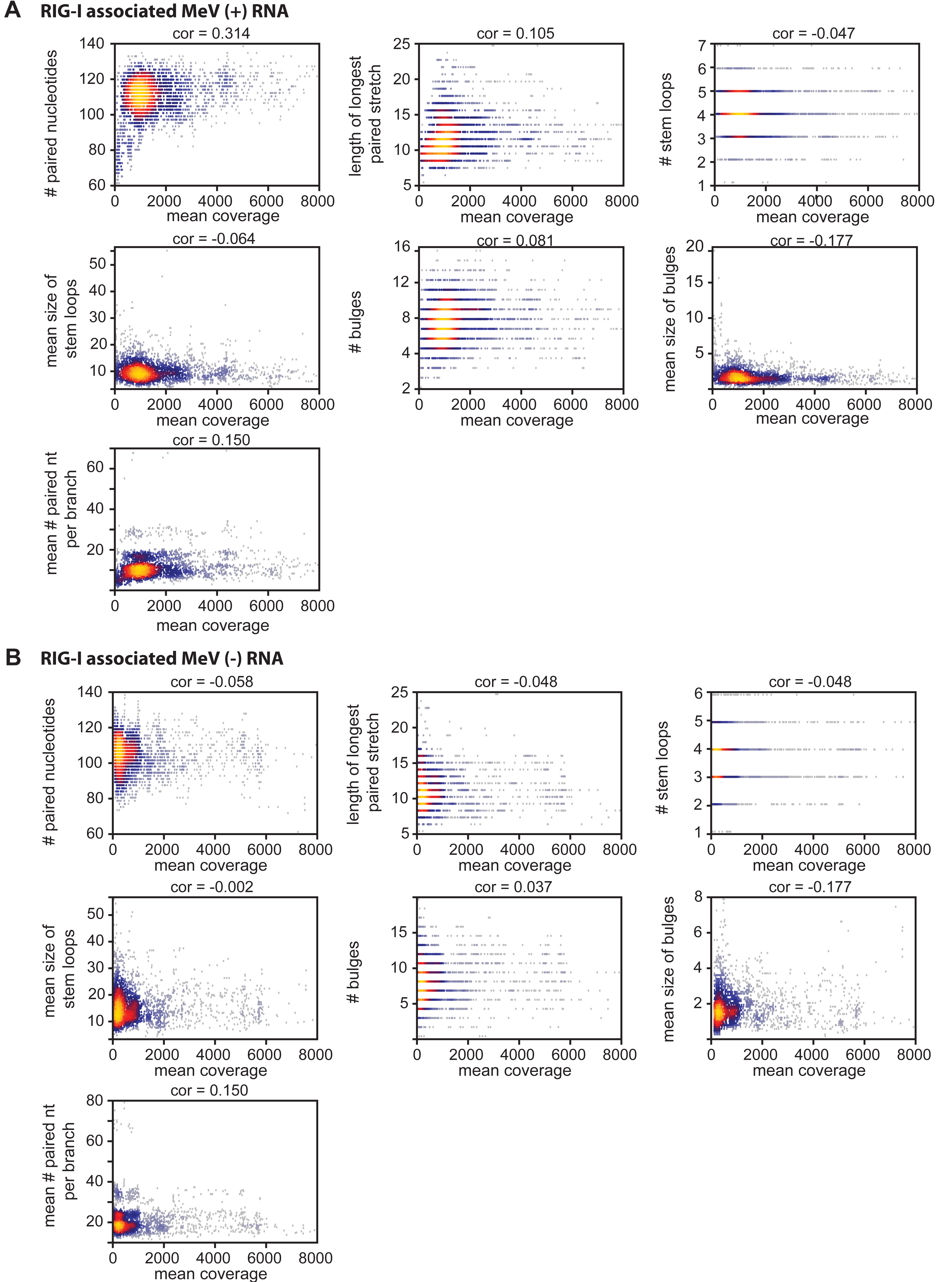

Supplement: Figure S5 — Secondary structure analysis of several features from in silico folded 201 nucleotide MeV RNA fragments and correlation to the fragment's mean coverage within the RIG-I sequencing library. In silico folding was done with RNAfold using standard parameters. The analysis is visualized in heatscatter plots and the linear correlation is expressed via the Pearson coefficient. Every dot corresponds to one fragment with its depicted feature and mean coverage. The more yellow the plot, the more data points overlap. Analyzed RNA features are: number of paired nucleotides, longest paired stretch, number of stem-loops, mean size of stem-loops, number of bulges, mean size of bulges and mean number of paired nucleotides per branch. A: Correlation analysis of RNA secondary structure features with the RIG-I associated RNA of positive polarity. B: Correlation analysis of RNA secondary structure features with the RIG-I associated RNA of negative polarity. (TIF) [file ppat.1004081.s005.tif]

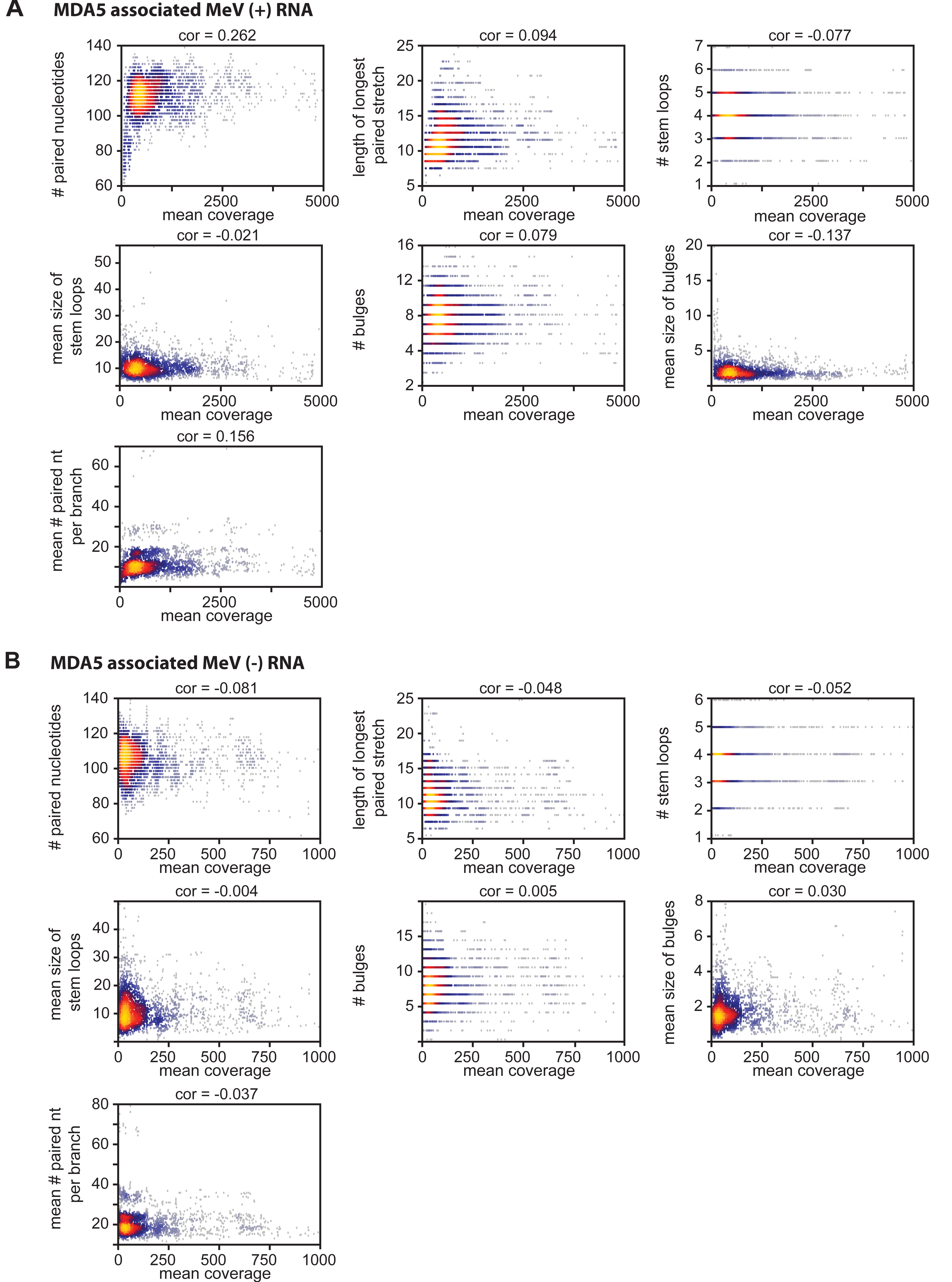

Supplement: Figure S6 — RNA secondary structure analysis of several features from in silico folded 201 nucleotide MeV RNA fragments and correlation to the fragment's mean coverage within the RIG-I sequencing library. Foldings were performed with RNAfold using standard parameters. The analysis is visualized in heatscatter plots and the linear correlation is expressed via the Pearson coefficient. Every dot corresponds to one fragment with its depicted feature and mean coverage. The more yellow the plot, the more data points overlap. Analyzed RNA features are: number of paired nucleotides, longest paired stretch, number of stem-loops, mean size of stem-loops, number of bulges, mean size of bulges and mean number of paired nucleotides per branch. A: Correlation analysis of RNA secondary structure features with the RIG-I associated RNA of positive polarity. B: Correlation analysis of RNA secondary structure features with the RIG-I associated RNA of negative polarity. (TIF) [file ppat.1004081.s006.tif]

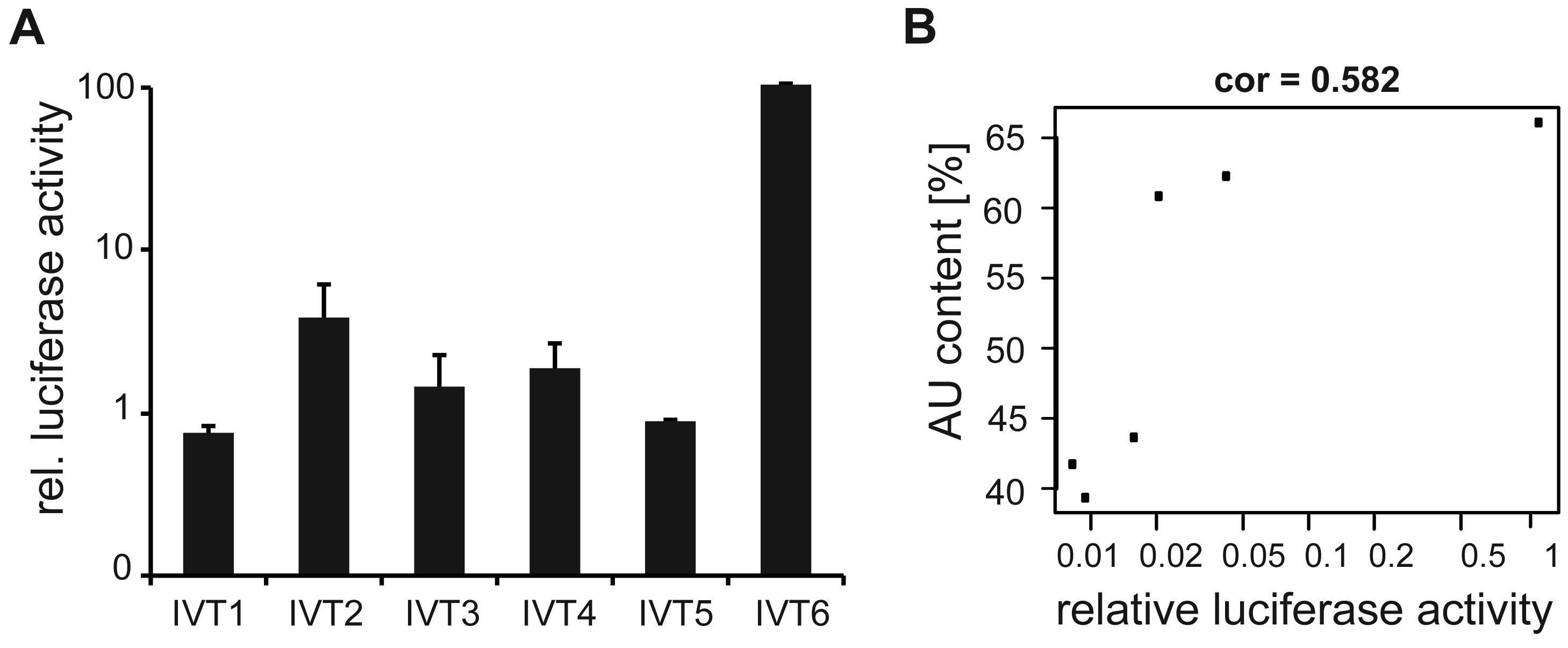

Supplement: Figure S7 — Analysis of in vitro transcribed RNA of the Mengo virus genome. Six 201 nt fragments were chosen to include low and high AU content. Transcripts were transfected into 293T ISRE-FF reporter cells in order to validate the immunostimulatory potential. A: Relative luciferase activity of transfected RNA (n = 3). B: Pearson correlation between (+) RNA maximal coverage and the relative luciferase activity. (TIF) [file ppat.1004081.s007.tif]

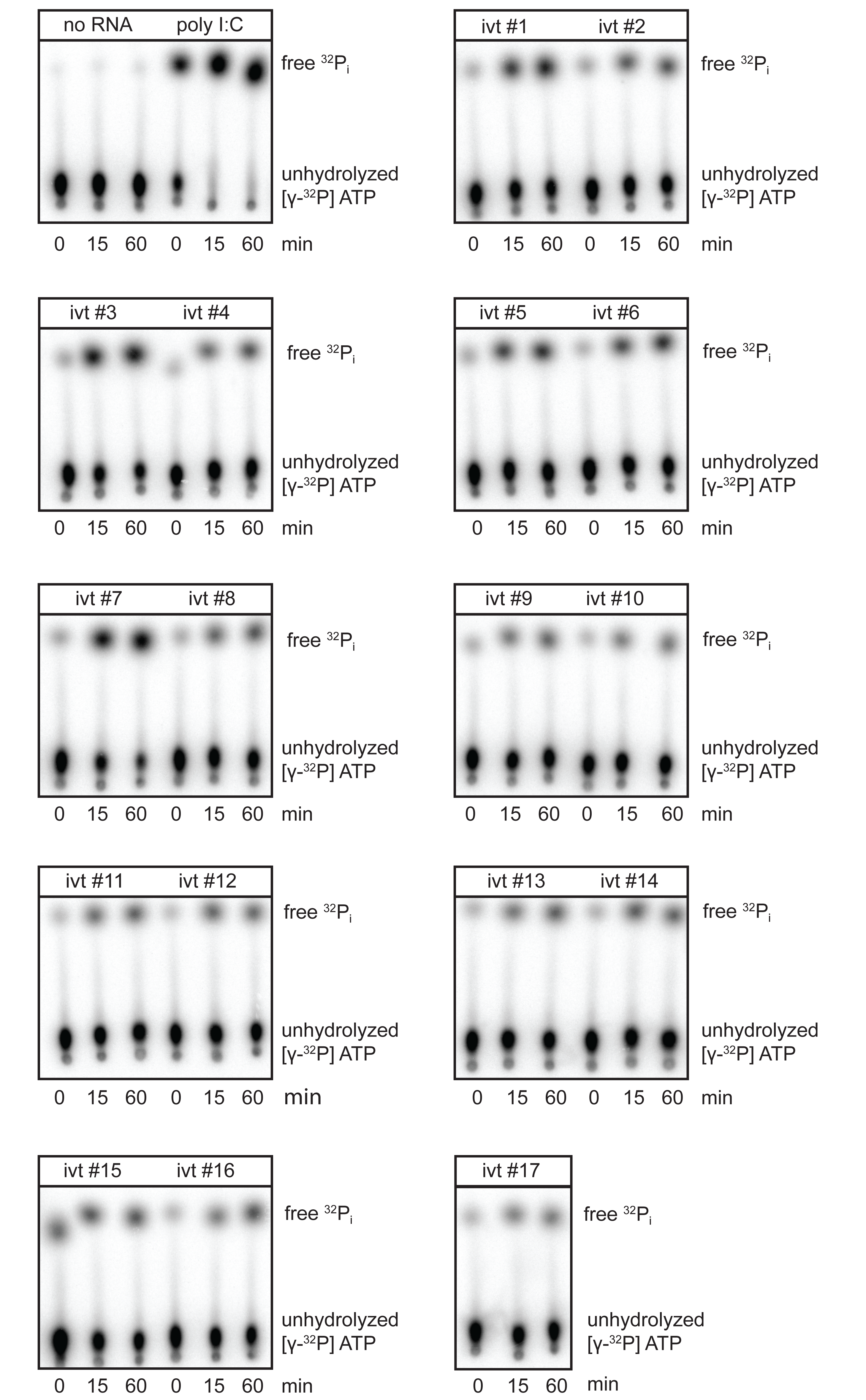

Supplement: Figure S8 — MDA5 ATPase activity assay. Free phosphate was separated by thin layer chromatography (TLC) and visualized on a Storm PhosphorImager from Molecular Dynamics. The ATPase hydrolysis rate was determined by quantifying free phosphate in comparison to non-hydrolyzed ATP 15 min after adding [γ-P32] ATP to the reaction mixture. (TIF) [file ppat.1004081.s008.tif]

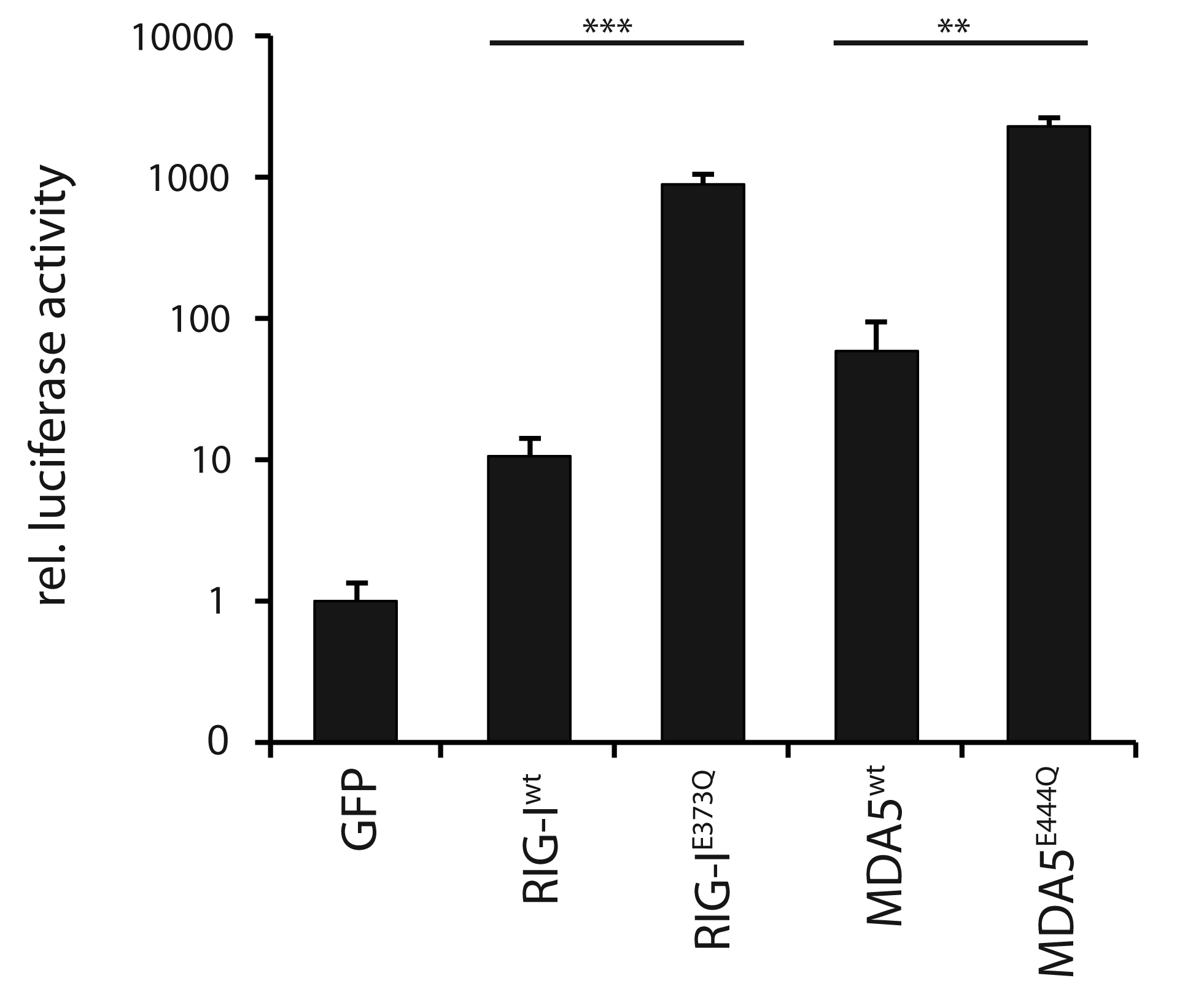

Supplement: Figure S9 — Immunostimulatory activity of overexpressed Walker B mutants RIG-IE373Q and MDA5E444Q compared to wildtype proteins in 293T ISRE-FF reporter cells (n = 3, ** P<0.01, *** P<0.001). (TIF) [file ppat.1004081.s009.tif]
