# Supplementary material for: In Vivo Ligands of MDA5 and RIG-I in Measles Virus-Infected Cells
Source: PLoS Pathog. 2014 Apr 17;10(4):e1004081. doi: 10.1371/journal.ppat.1004081 (PMC3990713; doi:10.1371/journal.ppat.1004081)
Supplement: Table S1 — Sequences of in vitro transcribed MeV RNAs. The gene annotation with the exact nucleotide position on the MeV genome is shown in brackets. (DOCX) [file ppat.1004081.s010.docx]

**Table S1:** **Sequences of *in vitro* transcribed MeV RNAs.** The gene annotation with the exact nucleotide position on the MeV genome is shown in brackets.

MeV IVT #1 (*le*N, 1-200)

ACCAAACAAAGUUGGGUAAGGAUAGUUCAAUCAAUGAUCAUCUUCUAGUGCACUUAGGAUUCAAGAUCCUAUUAUCAGGGACAAGAGCAGGAUUAGGGAUAUCCGAGAUGGCCACACUUUUAAGGAGCUUAGCAUUGUUCAAAAGAAACAAGGACAAACCACCCAUUACAUCAGGAUCCGGUGGAGCCAUCAGAGGAAUC

MeV IVT #2 (N, 201-400)

CAAACACAUUAUUAUAGUACCAAUCCCUGGAGAUUCCUCAAUUACCACUCGAUCCAGACUUCUGGACCGGUUGGUGAGGUUAAUUGGAAACCCGGAUGUGAGCGGGCCCAAACUAACAGGGGCACUAAUAGGUAUAUUAUCCUUAUUUGUGGAGUCUCCAGGUCAAUUGAUUCAGAGGAUCACCGAUGACCCUGACGUUA

MeV IVT #3 (P, 1746-1945)

CUUAGGAACCAGGUCCACACAGCCGCCAGCCCAUCAACCAUCCACUCCCACGAUUGGAGCCAAUGGCAGAAGAGCAGGCACGCCAUGUCAAAAACGGACUGGAAUGCAUCCGGGCUCUCAAGGCCGAGCCCAUCGGCUCACUGGCCAUCGAGGAAGCUAUGGCAGCAUGGUCAGAAAUAUCAGACAACCCAGGACAGGAG

MeV IVT #4 (P, 2601-2800)

CAGGUGCACCUGCGGGGAAUGUCCCCGAGUGUGUGAGCAAUGCCGCACUGAUACAGGAGUGGACACCCGAAUCUGGUACCACAAUCUCCCCGAGAUCCCAGAAUAAUGAAGAAGGGGGAGACUAUUAUGAUGAUGAGCUGUUCUCUGAUGUCCAAGAUAUUAAAACAGCCUUGGCCAAAAUACACGAGGAUAAUCAGAAG

MeV IVT #5 (M, 4601-4800)

AACAGCCCUGACACAAGGCCACCACCAGCCACCCCAAUCUGCAUCCUCCUCGUGGGACCCCCGAGGACCAACCCCCAAGGCUGCCCCCGAUCCAAACCACCAACCGCAUCCCCACCACCCCCGGGAAAGAAACCCCCAGCAAUUGGAAGGCCCCUCCCCCUCUUCCUCAACACAAGAACUCCACAACCGAACCGCACAAG

MeV IVT #6 (F, 5451-5650)

UGUCCAUCAUGGGUCUCAAGGUGAACGUCUCUGCCAUAUUCAUGGCAGUACUGUUAACUCUCCAAACACCCACCGGUCAAAUCCAUUGGGGCAAUCUCUCUAAGAUAGGGGUGGUAGGAAUAGGAAGUGCAAGCUACAAAGUUAUGACUCGUUCCAGCCAUCAAUCAUUAGUCAUAAAAUUAAUGCCCAAUAUAACUCUC

MeV IVT #7 (H, 8651-8850)

GAACCUAGCCUUAGGUGUAAUCAACACAUUGGAGUGGAUACCGAGAUUCAAGGUUAGUCCCUACCUCUUCACUGUCCCAAUUAAGGAAGCAGGCGAAGACUGCCAUGCCCCAACAUACCUACCUGCGGAGGUGGAUGGUGAUGUCAAACUCAGUUCCAAUCUGGUGAUUCUACCUGGUCAAGAUCUCCAAUAUGUUUUGG

MeV IVT #8 (L, 9801-10000)

GAGACACACACCUGUAUUCUUCACUGGUAGUUCAGUUGAGUUGCUAAUCUCUCGUGACCUUGUUGCUAUAAUCAGUAAAGAGUCUCAACAUGUAUAUUACCUGACAUUUGAACUGGUUUUGAUGUAUUGUGAUGUCAUAGAGGGGAGGUUAAUGACAGAGACCGCUAUGACUAUUGAUGCUAGGUAUACAGAGCUUCUAG

MeV IVT #9 (L, 10001-10200)

GAAGAGUCAGAUACAUGUGGAAACUGAUAGAUGGUUUCUUCCCUGCACUCGGGAAUCCAACUUAUCAAAUUGUAGCCAUGCUGGAGCCUCUUUCACUUGCUUACCUGCAGCUGAGGGAUAUAACAGUAGAACUCAGAGGUGCUUUCCUUAACCACUGCUUUACUGAAAUACAUGAUGUUCUUGACCAAAACGGGUUUUCU

MeV IVT #10 (L, 10201-10400)

GAUGAAGGUACUUAUCAUGAGUUAACUGAAGCUCUAGAUUACAUUUUCAUAACUGAUGACAUACAUCUGACAGGGGAGAUUUUCUCAUUUUUCAGAAGUUUCGGCCACCCCAGACUUGAAGCAGUAACGGCUGCUGAAAAUGUUAGGAAAUACAUGAAUCAGCCUAAAGUCAUUGUGUAUGAGACUCUGAUGAAAGGUCA

MeV IVT #11 (L, 10901-11100)

GCCAAGUGAUUGCUGAAAAUCUAAUCUCAAACGGGAUUGGCAAAUAUUUUAAGGACAAUGGGAUGGCCAAGGAUGAGCACGAUUUGACUAAGGCACUCCACACUCUAGCUGUCUCAGGAGUCCCCAAAGAUCUCAAAGAAAGUCACAGGGGGGGGCCAGUCUUAAAAACCUACUCCCGAAGCCCAGUCCACACAAGUACC

MeV IVT #12 (L, 11701-11900)

ACAAUUGUUUCAUCACAUUUUUUUGUCUAUUCAAAAGGAAUAUAUUAUGAUGGGCUACUUGUGUCCCAAUCACUCAAGAGCAUCGCAAGAUGUGUAUUCUGGUCAGAGACUAUAGUUGAUGAAACAAGGGCAGCAUGCAGUAAUAUUGCUACAACAAUGGCUAAAAGCAUCGAGAGAGGUUAUGACCGUUACCUUGCAUA

MeV IVT #13 (L, 12601-12800)

AGAAAUGUCCUCAUUGACAAAGAGUCAUGUUCAGUGCAGCUGGCGAGAGCUCUAAGAAGCCAUAUGUGGGCGAGGCUAGCUCGAGGACGGCCUAUUUACGGCCUUGAGGUCCCUGAUGUACUAGAAUCUAUGCGAGGCCACCUUAUUCGGCGUCAUGAGACAUGUGUCAUCUGCGAGUGUGGAUCAGUCAACUACGGAUG

MeV IVT #14 (L, 14501-14700)

GAAUCGGGUUGAACUCAUCUGCUUGCUACAAAGCUGUUGAGAUAUCAACAUUAAUUAGGAGAUGCCUUGAGCCAGGGGAGGACGGCUUGUUCUUGGGUGAGGGAUCGGGUUCUAUGUUGAUCACUUAUAAGGAGAUACUUAAACUAAACAAGUGCUUCUAUAAUAGUGGGGUUUCCGCCAAUUCUAGAUCUGGUCAAAGG

MeV IVT #15 (L, 14751-14950)

AGUAGGUAAUAUUGUCAAAGUGCUCUUUAACGGGAGGCCCGAAGUCACGUGGGUAGGCAGUGUAGAUUGCUUCAAUUUCAUAGUUAGUAAUAUCCCUACCUCUAGUGUGGGGUUUAUCCAUUCAGAUAUAGAGACCUUGCCUGACAAAGAUACUAUAGAGAAGCUAGAGGAAUUGGCAGCCAUCUUAUCGAUGGCUCUGC

MeV IVT #16 (L, 15101-15300)

AUUUGGUUAUGACAGAUCUCAAGGCUAACCGGCUAAUGAAUCCUGAAAAGAUUAAGCAGCAGAUAAUUGAAUCAUCUGUGAGGACUUCACCUGGACUUAUAGGUCACAUCCUAUCCAUUAAGCAACUAAGCUGCAUACAAGCAAUUGUGGGAGACGCAGUUAGUAGAGGUGAUAUCAAUCCUACUCUGAAAAAACUUACA

MeV IVT #17 (Ltr, 15696-1594)

UUUGAAACGUGAGUGGGUUUUUAAGGUAACAGUCAAGGAGACCAAAGAAUGGUAUAAGUUAGUCGGAUACAGUGCCCUGAUUAAGGACUAAUUGGUUGAACUCCGGAACCCUAAUCCUGCCCUAGGUGGUUAGGCAUUAUUUGCAAUAUAUUAAAGAAAACUUUGAAAAUACGAAGUUUCUAUUCCCAGCUUUGUCUGGU
