# Supplementary material for: In Vivo Ligands of MDA5 and RIG-I in Measles Virus-Infected Cells
Source: PLoS Pathog. 2014 Apr 17;10(4):e1004081. doi: 10.1371/journal.ppat.1004081 (PMC3990713; doi:10.1371/journal.ppat.1004081)
Supplement: Table S2 — Oligonucleotides used for generation of in vitro transcribed MeV sequences. (DOCX) [file ppat.1004081.s011.docx]

**Table S2: Oligonucleotides used for generation of *in vitro* transcribed MeV sequences.**

MeV IVT primer pair #1

fwd: GCGTAATACGACTCACTATAGGGACCAAACAAAGTTGGGTAA

rev: GATTCCTCTGATGGCTCCAC

MeV IVT primer pair #2

fwd: GCGTAATACGACTCACTATAGGGCAAACACATTATTATAGTACC

rev: TAACGTCAGGGTCATCGGTGA

MeV IVT primer pair #3

fwd: GCGTAATACGACTCACTATAGGGCTTAGGAACCAGGTCCACAC

rev: CTCCTGTCCTGGGTTGTCTGA

MeV IVT primer pair #4

fwd: GCGTAATACGACTCACTATAGGGCAGGTGCACCTGCGGGGAAT

rev: CTTCTGATTATCCTCGTGTAT

MeV IVT primer pair #5

fwd: GCGTAATACGACTCACTATAGGGAACAGCCCTGACACAAGGCC

rev: CTTGTGCGGTTCGGTTGTGGA

MeV IVT primer pair #6

fwd: GCGTAATACGACTCACTATAGGGTGTCCATCATGGGTCTCAAGG

rev: GAGAGTTATATTGGGCATTAA

MeV IVT primer pair #7

fwd: GCGTAATACGACTCACTATAGGGGAACCTAGCCTTAGGTGTAAT

rev: CCAAAACATATTGGAGATCTT

MeV IVT primer pair #8

fwd: GCGTAATACGACTCACTATAGGGGAGACACACACCTGTATTCTT

rev: CTAGAAGCTCTGTATACCTAG

MeV IVT primer pair #9

fwd: GCGTAATACGACTCACTATAGGGGAAGAGTCAGATACATGTGG

rev: AGAAAACCCGTTTTGGTCAAG

MeV IVT primer pair #10

fwd: GCGTAATACGACTCACTATAGGGGATGAAGGTACTTATCATGAG

rev: TGACCTTTCATCAGAGTCTCA

MeV IVT primer pair #11

fwd: GCGTAATACGACTCACTATAGGGGCCAAGTGATTGCTGAAAATC

rev: GGTACTTGTGTGGACTGGGCT

MeV IVT primer pair #12

fwd: GCGTAATACGACTCACTATAGGGACAATTGTTTCATCACATTTTT

rev: TATGCAAGGTAACGGTCATAA

MeV IVT primer pair #13

fwd: GCGTAATACGACTCACTATAGGGAGAAATGTCCTCATTGACAAA

rev: CATCCGTAGTTGACTGATCCA

MeV IVT primer pair #14

fwd: GCGTAATACGACTCACTATAGGGGAATCGGGTTGAACTCATCTG

rev: CCTTTGACCAGATCTAGAATT

MeV IVT primer pair #15

fwd: GCGTAATACGACTCACTATAGGGAGTAGGTAATATTGTCAAAGT

rev: GCAGAGCCATCGATAAGATGG

MeV IVT primer pair #16

fwd: GCGTAATACGACTCACTATAGGGATTTGGTTATGACAGATCTCA

rev: TGTAAGTTTTTTCAGAGTAGG

MeV IVT primer pair #17

fwd: GCGTAATACGACTCACTATAGGGTTTGAAACGTGAGTGGGTTTTT

rev: ACCAGACAAAGCTGGGAATAG
