# Supplementary material for: In Vivo Ligands of MDA5 and RIG-I in Measles Virus-Infected Cells
Source: PLoS Pathog. 2014 Apr 17;10(4):e1004081. doi: 10.1371/journal.ppat.1004081 (PMC3990713; doi:10.1371/journal.ppat.1004081)
Supplement: Table S3 — Sequences of in vitro transcribed Mengo RNAs. The gene annotation with the exact nucleotide position on the Mengo virus genome is shown in brackets. (DOCX) [file ppat.1004081.s012.docx]

**Table S3:** **Sequences of *in vitro* transcribed Mengo RNAs.** The gene annotation with the exact nucleotide position on the Mengo virus genome is shown in brackets.

Mengo IVT #1 (377-577)

GCGACCCUUUGCAGGCAGCGGAAUCCCCCACCUGGUGACAGGUGCCUCUGCGGCCGAAAGCCACGUGUGUAAGACACACCUGCAAAGGCGGCACAACCCCAGUGCCACGUUGUGCGUUGGAUAGUUGUGGAAAGAGUCAAAUGGCUCUCCUCAAGCGUAUUCAACAAGGGGCUGAAGGAUGCCCAGAAGGUACCCCACUGG

Mengo IVT #2 (803-1003)

UAUGAUGAAGAGUGGUACCCUGAGGAGUUGUUGACUGAUGGUGAAGAUGAUGUGUUCGAUCCUGAUUUGGACAUGGAAGUUGUGUUCGAGACACAAGGCAAUUCAACCUCAUCCGAUAAAAACAAUUCUUCUUCUGAGGGUAAUGAAGGAGUGAUUAUAAAUAAUUUCUAUUCCAACCAAUACCAAAAUUCAAUUGAUUUA

Mengo IVT #3 (2907-3107)

GUUUUGUCUCUUUUCCCCCUUUGUGUAUUAUAAAUGUGACCUGGAAGUGACCCUAAGUCCACACACCUCCGGUGCUCACGGGCUGUUGGUCCGCUGGUGCCCUACCGGAACUCCCACCAAGCCCACCACCCAGGUGCUGCAUGAGGUAAGUUCUCUCUCAGAAGGGCGAACCCCACAGGUGUACAGUGCCGGACCUGGUAC

Mengo IVT #4 (3103-3303)

UGGUACUUCCAAUCAGAUUUCAUUUGUAGUUCCUUAUAAUUCGCCUUUAUCUGUUCUGCCUGCUGUUUGGUAUAAUGGGCAUAAGAGAUUUGACAACACAGGCGACUUGGGAAUAGCUCCCAAUUCUGAUUUCGGCACCCUCUUCUUUGCUGGAACGAAACCUGAUAUUAAAUUCACUGUAUAUUUGAGAUACAAAAACAU

Mengo IVT #5 (6103-6303)

GGGCUUCCAUUCAGCCGGCUCCAUGGGCGUUGCAGCCGCGUCGAUAAUUUCACAAGAAAUGAUCGAUGCGGUGGUGCAGGCCUUCGAGCCCCAGGGUGCACUUGAGCGGCUGCCAGAUGGUCCGCGCAUCCAUGUACCCCGAAAGACUGCUUUGCGCCCGACUGUUGCCAGACAGGUCUUCCAACCCGCUUUUGCCCCAGC

Mengo IVT #6 (7095-8195)

UGUUAAAUACAAUAAUGAAUAAUAUUAUUAUUAGGGCCGGUUUGUAUCUUACAUAUAAAAAUUUUGAGUUUGAUGACGUGAAGGUCUUGUCUUAUGGUGAUGAUCUUCUAGUGGCAACUAAUUACCAAUUGAACUUUGAUAGAGUGAGAACAAGCCUGGCAAAGACAGGAUAUAAGAUUACACCCGCUAACAAAACUUCUA
