# Supplementary material for: In Vivo Ligands of MDA5 and RIG-I in Measles Virus-Infected Cells
Source: PLoS Pathog. 2014 Apr 17;10(4):e1004081. doi: 10.1371/journal.ppat.1004081 (PMC3990713; doi:10.1371/journal.ppat.1004081)
Supplement: Table S4 — Oligonucleotides used for generation of in vitro transcribed Mengo sequences. (DOCX) [file ppat.1004081.s013.docx]

**Table S4: Oligonucleotides used for generation of *in vitro* transcribed Mengo sequences.**

Mengo IVT primer pair #1

fwd: GCGTAATACGACTCACTATAGGGGCGACCCTTTGCAGG

rev: CCAGTGGGGTACCTTCTGGGC

Mengo IVT primer pair #2

fwd: GCGTAATACGACTCACTATAGGGTATGATGAAGAGTGG

rev: TAAATCAATTGAATTTTGGTA

Mengo IVT primer pair #3

fwd: GCGTAATACGACTCACTATAGGGGTTTTGTCTCTTTTCC

rev: GTACCAGGTCCGGCACTGTAC

Mengo IVT primer pair #4

fwd: GCGTAATACGACTCACTATAGGGTGGTACTTCCAATCAG

rev: ATGTTTTTGTATCTCAAATAT

Mengo IVT primer pair #5

fwd: GCGTAATACGACTCACTATAGGGGGGCTTCCATTCAGCCG

rev: GCTGGGGCAAAAGCGGGTTGG

Mengo IVT primer pair #6

fwd: GCGTAATACGACTCACTATAGGGTGTTAAATACAATAAT

rev: TAGAAGTTTTGTTAGCGGGTG
